# Dual targeting of a virus movement protein to ER and plasma membrane subdomains is essential for plasmodesmata localization

**DOI:** 10.1371/journal.ppat.1006463

**Published:** 2017-06-22

**Authors:** Kazuya Ishikawa, Masayoshi Hashimoto, Akira Yusa, Hiroaki Koinuma, Yugo Kitazawa, Osamu Netsu, Yasuyuki Yamaji, Shigetou Namba

**Affiliations:** Laboratory of Plant Pathology, Graduate School of Agricultural and Life Sciences, The University of Tokyo, 1-1-1 Yayoi, Bunkyo-ku, Tokyo, Japan; University of Kentucky, UNITED STATES

## Abstract

Plant virus movement proteins (MPs) localize to plasmodesmata (PD) to facilitate virus cell-to-cell movement. Numerous studies have suggested that MPs use a pathway either through the ER or through the plasma membrane (PM). Furthermore, recent studies reported that ER-PM contact sites and PM microdomains, which are subdomains found in the ER and PM, are involved in virus cell-to-cell movement. However, functional relationship of these subdomains in MP traffic to PD has not been described previously. We demonstrate here the intracellular trafficking of fig mosaic virus MP (MP_FMV_) using live cell imaging, focusing on its ER-directing signal peptide (SP_FMV_). Transiently expressed MP_FMV_ was distributed predominantly in PD and patchy microdomains of the PM. Investigation of ER translocation efficiency revealed that SP_FMV_ has quite low efficiency compared with SPs of well-characterized plant proteins, calreticulin and CLAVATA3. An MP_FMV_ mutant lacking SP_FMV_ localized exclusively to the PM microdomains, whereas SP chimeras, in which the SP of MP_FMV_ was replaced by an SP of calreticulin or CLAVATA3, localized exclusively to the nodes of the ER, which was labeled with *Arabidopsis* synaptotagmin 1, a major component of ER-PM contact sites. From these results, we speculated that the low translocation efficiency of SP_FMV_ contributes to the generation of ER-translocated and the microdomain-localized populations, both of which are necessary for PD localization. Consistent with this hypothesis, SP-deficient MP_FMV_ became localized to PD when co-expressed with an SP chimera. Here we propose a new model for the intracellular trafficking of a viral MP. A substantial portion of MP_FMV_ that fails to be translocated is transferred to the microdomains, whereas the remainder of MP_FMV_ that is successfully translocated into the ER subsequently localizes to ER-PM contact sites and plays an important role in the entry of the microdomain-localized MP_FMV_ into PD.

## Author summary

Intercellular trafficking of molecules through plasmodesmata (PD) is indispensable for plant development. Plant viruses also use the intercellular trafficking system to establish systemic infection. Virus movement proteins (MPs), which have abilities to localize to PD and to move to the adjacent cells autonomously, play important roles in facilitating virus cell-to-cell movement. Hence, understanding how MPs reach PD has great significance for virology and plant cell biology. In this study, we analyzed the intracellular trafficking of fig mosaic virus movement protein (MP_FMV_) mainly based on its N-terminal signal peptide (SP). SPs, short peptides directing proteins to the ER, are frequently found in a diverse array of proteins, but rarely found in plant virus proteins. We focused on the SP of MP_FMV_ and investigated the relationship between ER translocation and PD localization. We showed that the SP of MP_FMV_ had quite low translocation efficiency and contributes to generating two distinct populations. Each population localized to specialized subdomains of the ER and PM, and was essential for PD localization, indicating that these subdomains and PD are functionally related. Thus, our findings offer new insights into cell-to-cell movement in plants.

## Introduction

Plasmodesmata (PD), channels providing symplastic continuity of the ER and the plasma membrane (PM) between adjacent cells, play vital roles in intercellular communication in plants [1]. The ER and PM passing through PD are highly specialized to regulate PD permeability [2,3]. Plant viruses must pass through PD to establish systemic infection. To modify PD function and facilitate the cell-to-cell movement, viruses have PD-targeting proteins, the so-called movement proteins (MPs) [4]. Hence, understanding how MPs reach PD will provide insight into the mechanism underlying virus cell-to-cell movement.

MPs have been frequently proposed to use a membrane trafficking pathway either through the ER or through the PM to reach PD. Several viruses possess MPs that reportedly use endomembrane trafficking through the ER. These MPs are apparently associated with the ER and PD in infected cells [5–8] or in cells transiently expressing only MPs [9], even though the detailed mechanism by which these MPs traffic from the ER to PD is unclear. On the one hand, other MPs such as those of cauliflower mosaic virus and cowpea mosaic virus localize to the PM [10,11]. Inhibition by brefeldin A (BFA), an inhibitor of COPII transport, showed that the secretory pathway is not involved in the PM localization [10,11], but how these MPs traffic to the PM is still unknown. One recent study has proposed that cauliflower mosaic virus MP is transported from the PM to PD through the endocytic pathway [12].

Recent studies have shown that two membrane subdomains in the ER and PM are also involved in virus cell-to-cell movement. ER-PM contact sites, membrane subdomains connecting between the cortical ER and the PM, are known to play roles in intracellular Ca^2+^ homeostasis and signaling in mammalian cells [13]. *Arabidopsis* synaptotagmin 1 (SYTA), a key component in connecting the cortical ER and the PM in plant cells [14,15], substantially localizes to the nodes of the cortical ER [15]. SYTA interacts with several virus MPs, and knockout or dominant-negative inhibition of SYTA delays cell-to-cell movement of several viruses [16,17]. These facts indicate that the function of ER-PM contact sites is important for virus cell-to-cell movement.

PM microdomains, small regions which have compositions and functions distinct from the surrounding PM, are another type of membrane subdomains that are involved in virus cell-to-cell movement. PM microdomains have been proposed to have lipid compositions that differ from the surrounding PM and to be detergent insoluble, although this is still a matter of debate [18]. The number and the biological roles of microdomains are largely unknown in plant cells, but certain proteins show patchy distribution in the PM and are recognized as microdomain-associated proteins [19]. One of the microdomain-associated proteins, remorin (REM1.3), suppresses cell-to-cell movement of potato virus X (PVX) [20]. Furthermore, REM1.3 localizes also to PD and interacts with triple gene block protein 1 (TGBp1), an MP of PVX. Thus, it has been suggested that PM microdomains as well as ER-PM contact sites are important for virus cell-to-cell movement. Considering that PD localization of virus MPs is necessary for facilitating virus cell-to-cell movement, these two membrane subdomains, microdomains and ER-PM contact sites, are speculated to be involved in MP traffic to PD [16,17,20]. However, there is no direct evidence that virus MPs use these subdomains to reach PD, and a functional relationship of these subdomains in MP traffic to PD is unclear.

Signal peptides (SPs) are short sequences comprising approximately 7–30 aa that are frequently found in the N terminus of a diverse array of proteins. In general, proteins in eukaryotic cells with an SP are co-translationally recruited to the ER, and penetrate the ER membrane or are released into the ER lumen concomitant with the SP cleavage [21,22]. Some of these proteins play specific roles in the ER, whereas others are further transported to other organelles or secreted into the extracellular space. Thus, SPs are essential for proper localization and membrane targeting of proteins.

*Fig mosaic virus* (FMV) is a negative-strand RNA virus in the genus *Emaravirus*. We showed previously that the MP of FMV (MP_FMV_) localized to the PM in addition to PD, and remarkably, MP_FMV_ was predicted to possess an N-terminal SP [23]. To the best of our knowledge, no viruses, other than the members of the genus *Emaravirus*, have an MP possessing an SP. In this study, we analyzed the intracellular trafficking of MP_FMV_ focusing on the SP function in hopes of determining how the recruitment of a virus MP to the ER is involved in PD targeting. As a result, we found that the SP of MP_FMV_ has extremely low ER translocation efficiency compared with conventional SPs of plant proteins, thereby causing abortive ER translocation of MP_FMV_ at a high frequency. A fraction of MP_FMV_ was translocated to the ER, whereas the remainder of MP_FMV_, which was not translocated to the ER, was transported to the patchy microdomains in the PM. Moreover, the ER-translocated MP_FMV_ specifically localized to the ER-PM contact sites and played an essential role in the entry of microdomain-localized MP_FMV_ into PD. Taken together, these findings suggest that dual targeting to two distinct subdomains in the ER and PM is essential for PD localization of MP_FMV_.

## Results

### MP_FMV_ was distributed predominantly in PD and the patchy microdomains of the PM

**Fig 1:**
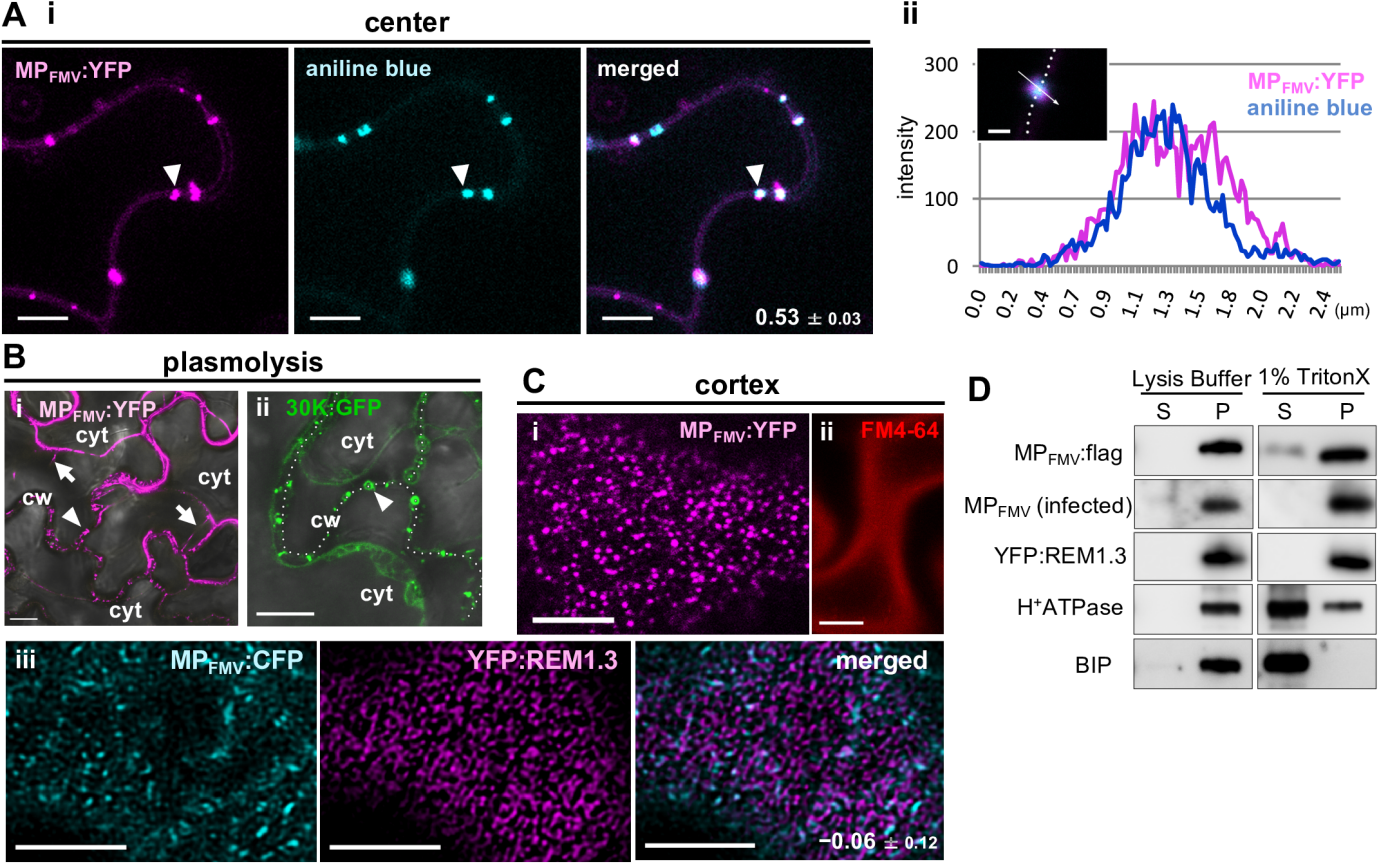
Investigation of the subcellular distribution of MP_FMV_:YFP. (A–C) Confocal imaging of MP_FMV_:YFP-expressing epidermal cells at 36 hours post-infiltration (hpi). YFP fluorescence was pseudocolored by magenta. (A) (i) Cells expressing MP_FMV_:YFP were treated with aniline blue. Arrowheads indicate plasmodesma. The mean ± SD of Pearson correlation coefficient (PCC [–1:1]) is given in the image. Bars = 5 μm. (ii) Fluorescence intensity along the arrow across plasmodesma. The dotted line in the confocal image indicates the cell wall. Bar = 1 μm. (B) Plasmolyzed cells expressing (i) MP_FMV_:YFP or (ii) 30K:GFP. Arrows indicate Hechtian strands extended from the PM. Arrowheads indicate plasmodesmata. The dotted line indicates the cell wall. cw, cell wall; cyt, cytoplasm. Bars = 10 μm. (C) Surface views of cells (i) expressing MP_FMV_:YFP or (ii) treated with FM4-64. (iii) Co-expression of MP_FMV_:CFP and YFP:REM1.3. To obtain higher resolution images, images were processed by a deconvolution algorithm. Bars = 5 μm. (D) 1% TritonX-100 treatment of membranes. Membrane-enriched fractions prepared from FMV-infected fig leaves and *N*. *benthamiana* leaves expressing MP_FMV_:FLAG or YFP:REM1.3 at 36 hpi were treated with 1% TritonX-100. Anti-FLAG, anti-MP_FMV_, anti-GFP, anti-H^+^ATPase and anti-BIP antibodies were used for the detection of MP_FMV_:FLAG, MP_FMV_, YFP:REM1.3, a PM marker H^+^ATPase and an ER marker BIP. S; soluble fraction. P; insoluble fraction.

MP_FMV_ was fused to YFP (MP_FMV_:YFP) to investigate its subcellular localization in *Nicotiana benthamiana*. Consistent with our previous results [23], transiently expressed MP_FMV_:YFP localized to the punctate structures along the PM in epidermal cells (Fig 1Ai). Treatment with aniline blue, which stains callose structures including PD, showed that the punctate structures of MP_FMV_:YFP colocalized with PD (Pearson correlation coefficient [PCC] = 0.53 ± 0.03). Measurement of the fluorescent intensity across plasmodesma shows that the fluorescence signal of MP_FMV_:YFP coincided with that of aniline blue (Fig 1Aii), showing that MP_FMV_:YFP localized to PD.

MP_FMV_:YFP appeared to accumulate also on the PM. PM and PD localization can be easily distinguished in plasmolyzed cells because PM proteins are associated with Hechtian strands, which are stretched PMs connecting the retracted PM and the cell wall, whereas PD proteins are retained in PD even during plasmolysis [24]. In plasmolyzed cells, MP_FMV_:YFP fluorescence was observed in Hechtian strands and PD (Fig 1Bi). On the other hand, fluorescence signal was observed only in PD, but not in Hechtian strands, when cells expressing tobacco mosaic virus MP as a GFP fusion (30K:GFP) were plasmolyzed (Fig 1Bii). These results indicate that MP_FMV_:YFP accumulated in the PM, in addition to PD. Observation of the cell surface revealed that MP_FMV_:YFP shows patchy distribution throughout the PM (Fig 1Ci). This distribution pattern was different from the fluorescence pattern of the PM stained with FM4-64, an amphiphilic styryl dye which is inserted into the outer layer of the PM (Fig 1Cii). These images were taken in the abaxial surface of the abaxial epidermal cells, and PD were not contained in these patches. Since such uneven distribution in the PM is similar to the localization of microdomain-associated proteins remorines [19,20], a YFP fusion with *A*. *thaliana* REM1.3 (YFP:REM1.3) was co-expressed with MP_FMV_:CFP; however, YFP:REM1.3 did not substantially co-localize with MP_FMV_:CFP (PCC = −0.06 ± 0.12; Fig 1Ciii). MP_FMV_ probably localized to the PM subdomains distinct from those of REM1.3.

To corroborate the subcellular distribution of MP_FMV_ observed by microscopy, we performed chemical treatment using 1% TritonX-100. 1% TritonX-100 was expected to solubilize proteins associated with the cellular membranes excluding proteins localized to detergent-insoluble domains, including PM microdomains [25]. As expected, TritonX-100 treatment solubilized the majority of a PM marker H^+^ATPase and an ER marker BIP (Fig 1D). Conversely, only a small proportion of transiently expressed MP_FMV_:FLAG was soluble in 1% TritonX-100. In FMV-infected cells, almost all MP_FMV_ was detected from insoluble fraction. MP_FMV_ became more insoluble in FMV-infected cells probably due to other virus factors. Furthermore, this result was in agreement with that of YFP:REM1.3, which localizes to the Triton-insoluble microdomains [19,20]. Given that MP_FMV_ unevenly distributed in the PM and that did not colocalize with REM1.3 (Fig 1Ciii), MP_FMV_ may localize to the detergent-insoluble microdomains different from those of REM1.3.

### MP_FMV_ has an ER-directing signal peptide at the N-terminus

**Fig 2:**
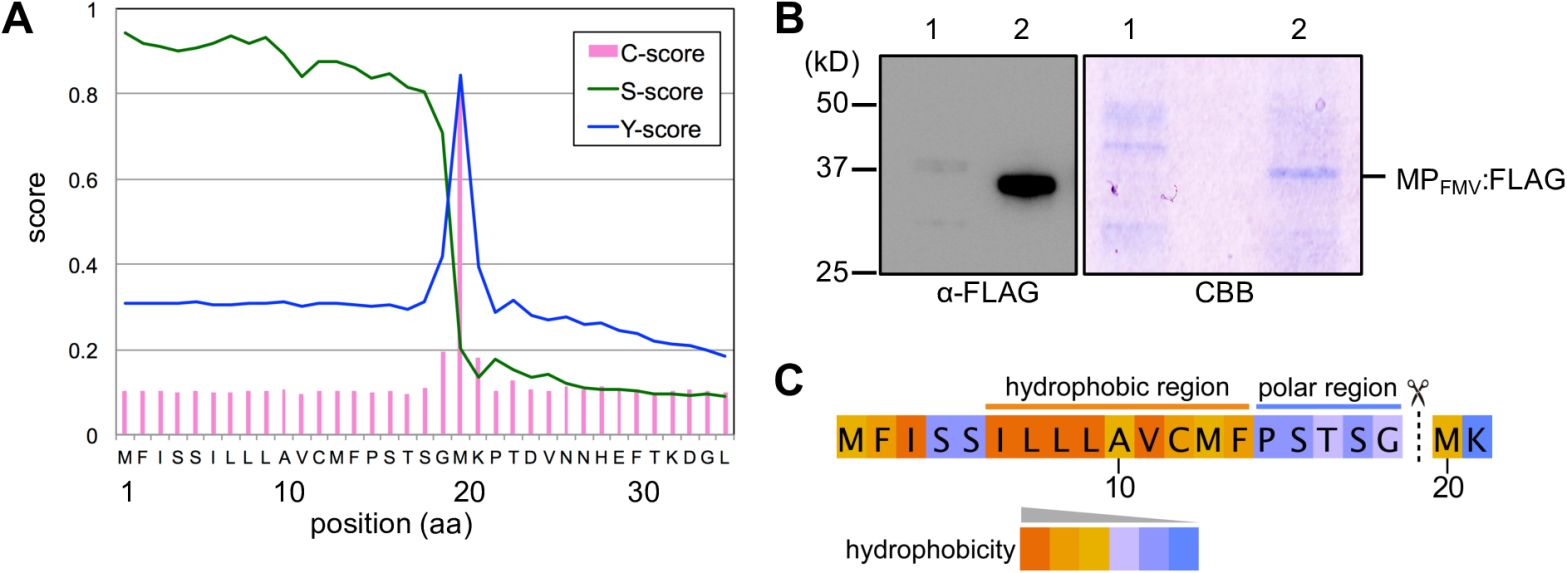
MP_FMV_ has an N-terminal signal peptide. (A) SignalP ver 4.1 predicted an N-terminal signal peptide in the MP_FMV_ sequence. C-score is the predicted cleavage site value, S-score is the predicted signal peptide value and Y-score is the cleavage site value calculated by combining C- and S-scores. (B) Immunoprecipitation of MP_FMV_:FLAG. MP_FMV_:FLAG was immunoprecipitated with anti-FLAG antibody from cell lysates of healthy control leaves (lane 1) or of MP_FMV_:FLAG expressing leaves at 48 hpi (lane 2). The immunoprecipitated samples were checked by immunoblot analysis using anti-FLAG antibody (left panel) and Coomassie Brilliant Blue (CBB) staining (right panel). The lane between lane 1 and 2 is blank in CBB staining. (C) Hydrophobicity of the predicted SP sequence. Amino acid residues are color-coded according to their hydrophobicity. The dotted line with scissors indicates the putative cleavage site.

An ER-directed SP was predicted at the N-terminus of MP_FMV_ by SignalP software in our previous work [23]. The cleavage site was between G19 and M20, and the length of the deduced SP was 19 aa (Fig 2A). To verify the cleavage at the predicted site, MP_FMV_:FLAG expressed transiently in *N*. *benthamiana* was purified by immunoprecipitation using anti-FLAG antibody. Immunoblot analysis using anti-FLAG antibody and Coomassie Brilliant Blue (CBB) staining of the immunoprecipitated samples showed a protein band of approximately 37 kD, indicating that precipitated MP_FMV_:FLAG has a single molecular weight (Fig 2B). Amino acid sequences beginning at M20, but not at the N-terminal methionine, were found in the sequences determined by Edman degradation of the purified MP_FMV_:FLAG, which means that the N-terminal 19 aa was cleaved off (S1 Fig). Combined with CBB staining and immunoblot analysis, the N-terminal 19 aa was suggested to be almost perfectly processed from MP_FMV_:FLAG. The N-terminal 19 aa had characteristics of SPs, a central hydrophobic region which forms an α-helix and a polar C-terminal region (Fig 2C) [21,22]. These results suggest that MP_FMV_ has an N-terminal SP (hereafter referred to as SP_FMV_), which is expected to translocate the nascent protein into the ER.

### SP_FMV_ has extremely low translocation efficiency

**Fig 3:**
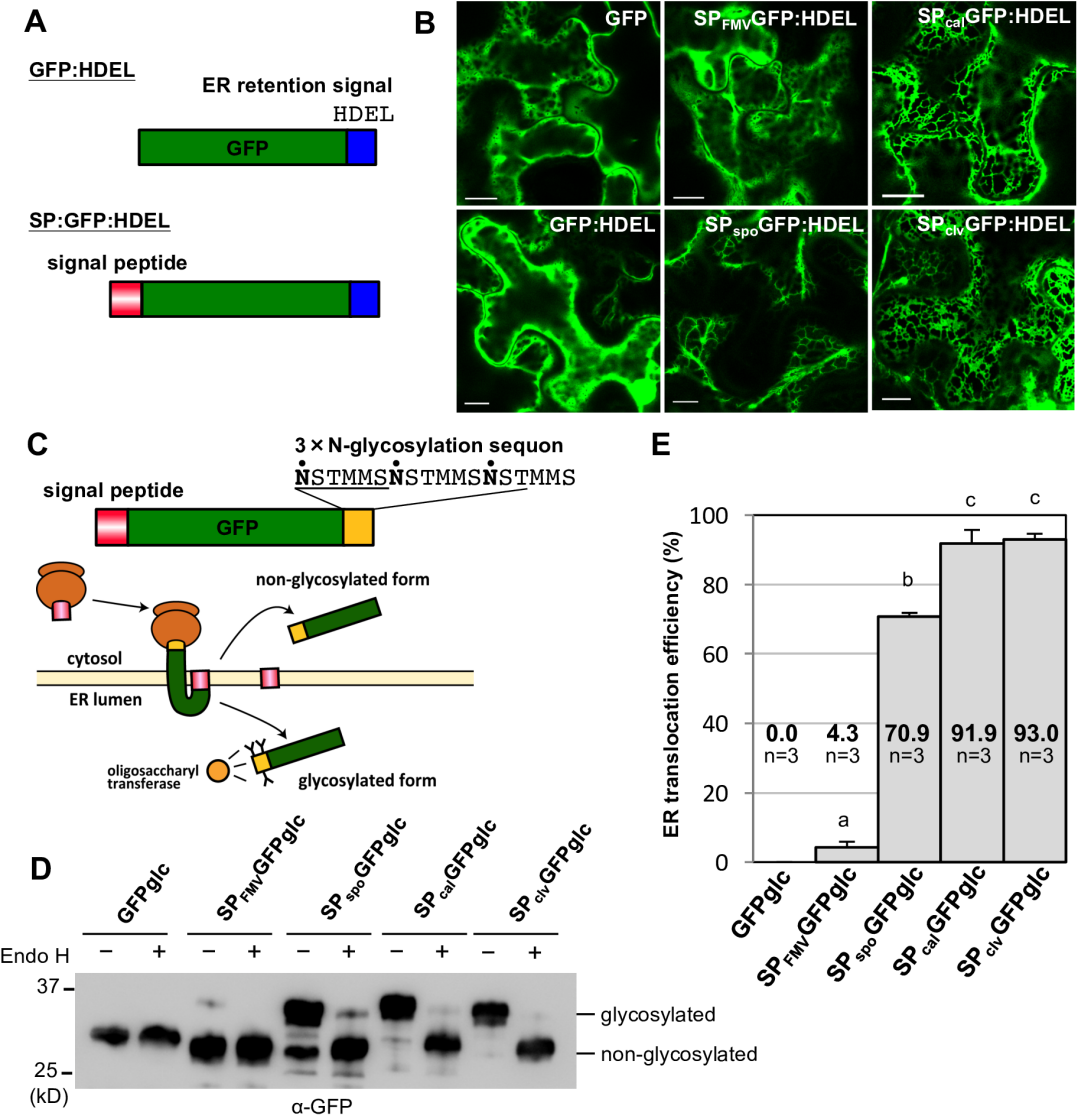
ER translocation efficiency of SP_FMV_ differs from those of plant protein SPs. (A) Schematic representation of fusion proteins for investigating ER translocation efficiency. (B) Cells expressing free GFP, GFP:HDEL, SP_FMV_GFP:HDEL, SP_spo_GFP:HDEL, SP_cal_GFP:HDEL and SP_clv_GFP:HDEL were observed at 36 hpi. Bars = 10 μm. (C) Schematic representation of the experimental system and fusion proteins with a 3×N-glycosylation sequon. Dots indicate asparagine residues expected to be glycosylated. SPs of MP_FMV_, sporamin A, calreticulin or CLAVATA3 were fused to the N terminus of GFP:glc, GFP carrying a 3×N-glycosylation sequon in its C terminus (SP_FMV_GFPglc, SP_spo_GFPglc, SP_cal_GFPglc and SP_clv_GFPglc, respectively). (D) Immunoblot analysis of proteins extracted from cells expressing GFPglc, SP_FMV_GFPglc, SP_spo_GFPglc, SP_cal_GFPglc or SP_clv_GFPglc using anti-GFP antibody. Samples were collected at 30 hpi. Glycosylation of each sample was confirmed by deglycosylation with Endo H. (E) Quantitation of translocation efficiencies. The bars show means + SD of three independent experiments. Different letters on the error bars indicate statistical differences at the 1% level of significance (Tukey test).

To confirm whether or not SP_FMV_ translocates a protein into the ER lumen as those of plant proteins, we constructed GFPs flanked with the N-terminal SPs derived from MP_FMV_ or plant proteins (sporamin A, calreticulin or CLAVATA3) and a C-terminal ER retention signal (Fig 3A; SP_FMV_GFP:HDEL, SP_spo_GFP:HDEL, SP_cal_GFP:HDEL and SP_clv_GFP:HDEL, respectively). We selected these three SPs because the SP activity was empirically assessed in previous studies [26–28]. These four SPs including SP_FMV_ have no sequence homology (S2 Fig). As controls, we prepared free GFP and GFP:HDEL. Here, it is noted that GFP:HDEL does not have an N-terminal SP. Unexpectedly, transiently expressed SP_FMV_GFP:HDEL was distributed throughout the cytosol, but not to the ER (Fig 3B). The fluorescence pattern appeared to be the same as that of free GFP or GFP:HDEL. In contrast, SP_spo_GFP:HDEL, SP_cal_GFP:HDEL and SP_clv_GFP:HDEL predominantly localized in the ER, as expected. These results indicate the functional difference in ER translocation between SP_FMV_ and the other SPs.

We further investigated the ER translocation ability of these SPs using N-glycosylation as an indicator. We constructed fusion proteins in which the 3× N-glycosylation sequon was C-terminally fused to GFP, SP_FMV_GFP, SP_spo_GFP, SP_cal_GFP and SP_clv_GFP (Fig 3C; GFPglc, SP_FMV_GFPglc, SP_spo_GFPglc, SP_cal_GFPglc and SP_clv_GFPglc, respectively) [29]. If an SP successfully translocates the GFP fusion into the ER lumen after SP cleavage by an ER membrane-bound SP peptidase on the lumenal side, glycosylation of asparagine residues in the sequon by an ER-resident enzyme, oligosaccharyl transferase, occurs and results in an increase in the molecular weight of the translocated GFPglc relative to that of the non-translocated GFPglc. Immunoblot analysis of total proteins extracted from leaves expressing GFPglc, SP_FMV_GFPglc, SP_spo_GFPglc, SP_cal_GFPglc or SP_clv_GFPglc using anti-GFP antibody showed that almost all of the SP_cal_GFPglc or SP_clv_GFPglc molecules were glycosylated (91.9% and 93.0%, respectively), and that more than half (70.9%) of the SP_spo_GFPglc molecules were glycosylated (Fig 3D and 3E). However, compared with these measurements, a dramatically lower proportion (4.3%) of the SP_FMV_GFPglc molecules were glycosylated. No glycosylation was detected in GFPglc, which lacks an SP. Thus, SP_FMV_ had much lower translocation efficiency compared with conventional SPs, suggesting that only a small proportion of MP_FMV_ molecules were translocated to the ER. In the case of SP_FMV_GFP:HDEL (Fig 3B), the fluorescence of ER-translocated GFP was thought to be masked by GFP fluorescence in the cytosol because SP_FMV_ translocated only a small fraction of GFP molecules into the ER.

### The subcellular localization pattern of MP_FMV_ is altered depending on the SP

To gain insight into the role of ER translocation in the intracellular trafficking of MP_FMV_, we constructed an MP_FMV_ mutant whose SP was not expected to be cleaved (ncMP). In this mutant, two substitutions, L7P and V11P, were introduced into the central α-helix region of SP_FMV_ to break the helix [21]. We verified that an SP was no longer predicted in the ncMP sequence by SignalP (S3A Fig). Transiently expressed ncMP as a YFP fusion (ncMP_FMV_:YFP) showed aberrant accumulation in the cytoplasm (S3Bi Fig), and did not target to the ER, PM and PD (S3Bii Fig). This result indicates that the cleavage of SP_FMV_ is essential for MP_FMV_ to localize properly.

**Fig 4:**
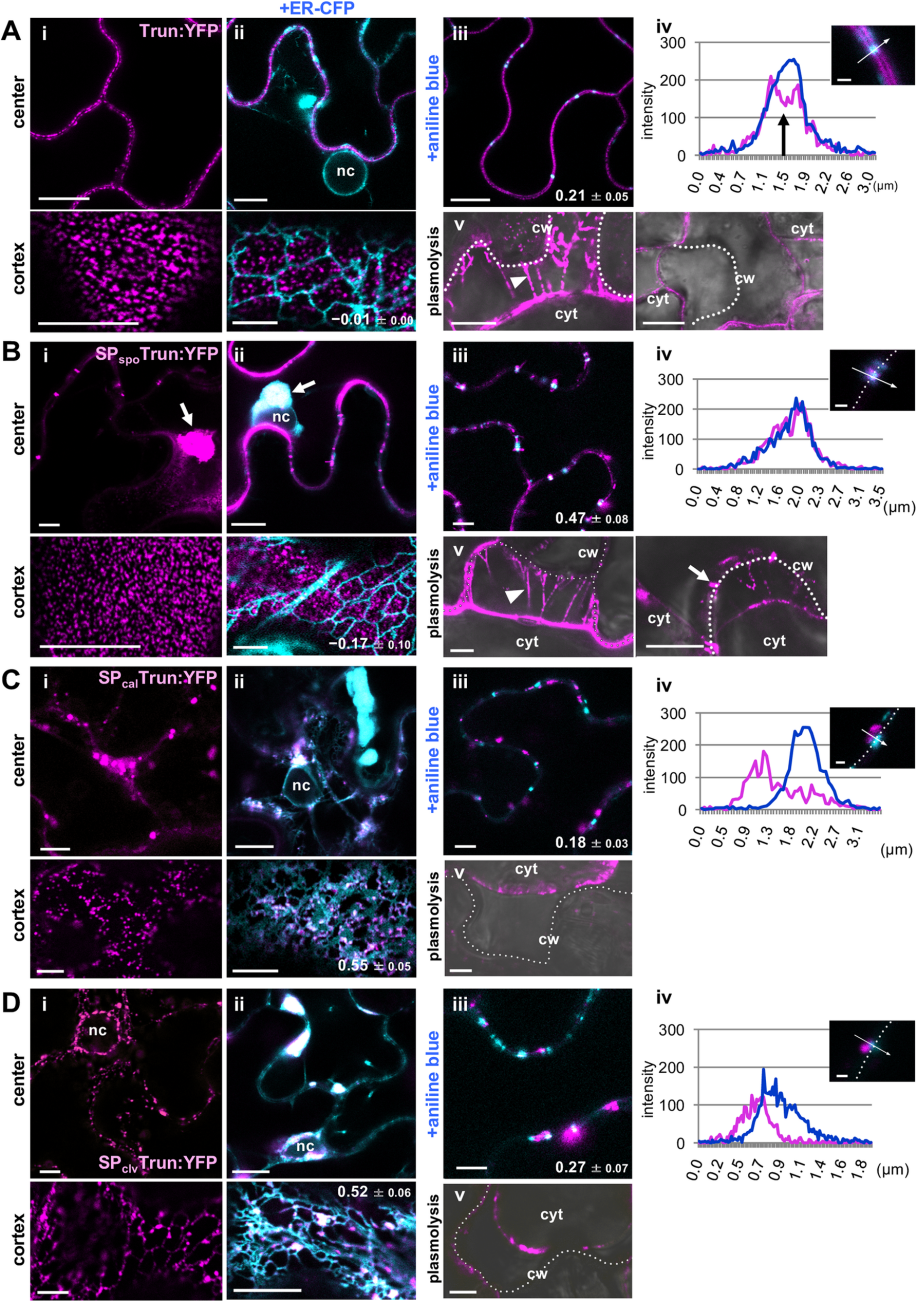
Deletion or replacement of SP_FMV_ alters the subcellular distribution of MP_FMV_. Localization analysis of (A) SP-deficient MP_FMV_, Trun:YFP, and SP chimeras; (B) SP_spo_Trun:YFP, (C) SP_cal_Trun:YFP and (D) SP_clv_Trun:YFP. Images through the center or through the cortex of cells expressing (i) Trun:YFP, SP_spo_Trun:YFP, SP_cal_Trun:YFP and SP_clv_Trun:YFP alone or (ii) with ER-CFP. Arrows indicate aggregations of SP_spo_Trun:YFP in the ER. nc; nucleus. (iii) Aniline blue treatment. (iv) Fluorescence intensity along the arrows across PD. A black arrow indicates the signal reduction of Trun:YFP in the center of PD. Dotted lines in the confocal image indicate the cell wall. Bar = 1 μm. (v) Plasmolysis. Bright-field images were merged. The dotted lines indicate the cell wall. Arrowheads indicate Hechtian strands extended from the PM (The left panels in A and B). The white arrow indicates fluorescence signals retained in PD (The right panels in B). cw, cell wall; cyt, cytoplasm. All images were captured at 36 hpi. YFP fluorescence is pseudocolored with magenta. Bars: (i)–(iii) and (v), 10 μm; (iv), 1 μm.

We assessed localization of an MP_FMV_ mutant lacking the N-terminal 19 aa SP. The SP-deficient mutant was fused with YFP (Trun:YFP) and expressed in the same conditions as MP_FMV_:YFP in Fig 1. Trun:YFP was distributed to the PM microdomains, similar to MP_FMV_:YFP (Fig 4Ai, compared with Fig 1Ci). The PM localization of Trun:YFP was checked by the fluorescence in Hechtian strands of plasmolyzed cells as was the case for MP_FMV_:YFP (Fig 4Av left panel). Co-expression with ER-CFP showed that Trun:YFP was not associated with the perinuclear or peripheral ER (PCC = −0.01 ± 0.00; Fig 4Aii). Also, Trun:YFP did not specifically localize to aniline blue-stained PD (PCC = 0.21 ± 0.05; Fig 4Aiii). The fluorescent signal of Trun:YFP was reduced in the region corresponding to the center of PD (Fig 4Aiv), unlike MP_FMV_:YFP (Fig 1Aii). Furthermore, we confirmed that Trun:YFP was not retained in PD in plasmolyzed cells (Fig 4Av right panel). These results indicate that the SP-deficient mutant did not localize to PD and that the SP_FMV_ is essential for the PD localization of MP_FMV_, but dispensable for targeting the PM microdomains.

Next, SP_FMV_ function was compared with SPs derived from plant proteins, sporamin A, calreticulin or CLAVATA3, by analyzing localization of SP chimeras in which the SPs of these proteins were fused to the N-terminus of Trun:YFP (SP_spo_Trun:YFP, SP_cal_Trun:YFP and SP_clv_Trun:YFP, respectively). SP_spo_Trun:YFP localized to the PM microdomains (Fig 4Bi, 4Bii and 4Bv) and PD (PCC = 0.47 ± 0.08; Fig 4Biii and 4Biv), similar to MP_FMV_:YFP. However, unlike MP_FMV_:YFP, cytoplasmic aggregations were observed in the SP_spo_Trun:YFP-expressing cells (Fig 4Bi). Co-expression with the ER marker ER-CFP showed that these aggregations were formed in the ER (Fig 4Bii). On the other hand, SP_cal_Trun:YFP and SP_clv_Trun:YFP were associated with nodes in the ER network (PCC: 0.55 ± 0.05 and 0.52 ± 0.06, respectively; Fig 4Ci, Cii, 4Di and 4Dii). Although some of these punctate spots were located in close proximity to PD, many of them did not co-localize with PD (PCC: 0.18 ± 0.03 and 0.27 ± 0.07, respectively; Fig 4Ciii and 4Diii). Fluorescence intensity measurements confirmed that fluorescence signals of SP_cal_Trun:YFP and SP_clv_Trun:YFP did not coincide with that of aniline blue (Fig 4Civ and 4Div). Hechtian strands were invisible when cells expressing SP_cal_Trun:YFP or SP_clv_Trun:YFP were plasmolyzed (Fig 4Cv and 4Dv), indicating that these chimeras were not distributed to the PM. These observations suggest that SP_FMV_ plays an essential role in MP_FMV_ localization.

### PD localization is necessary for exerting MP_FMV_ functions

**Fig 5:**
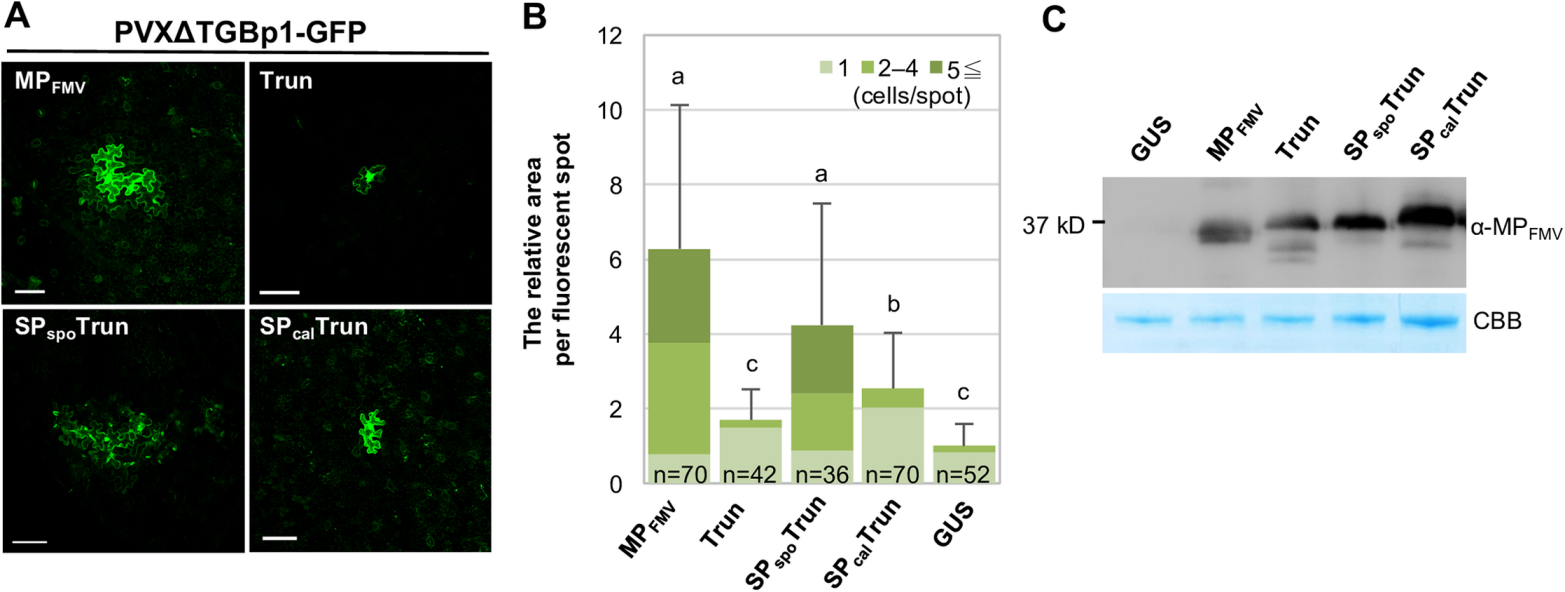
PD localization of MP_FMV_ is necessary to facilitate virus cell-to-cell movement. TGBp1-deficient PVX (PVXΔTGBp1-GFP) was co-expressed with GUS, MP_FMV_ or its mutants (Trun, SP_spo_Trun or SP_cal_Trun). (A) Typical images were captured at 5 days post-infiltration (dpi). Bars = 100 μm. (B) Quantification analysis. Measurements were normalized with GUS control; the mean value of GUS-expressing leaves was taken as 1.0. The bars show means + SD. n indicates the total number of measurements in two independent experiments. Different letters on error bars indicate statistical differences at the 1% level of significance (Steel-Dwass test). The fluorescence area is categorized by the cell number per fluorescent spot and shown by different colors. (C) Immunoblot analysis using anti-MP_FMV_ antibody (top panel) confirmed expression of MP_FMV_ and MP_FMV_ mutants. CBB staining is shown as a loading control (bottom panel). Samples were collected at 36 hpi.

Our previous study showed that the expression of MP_FMV_ complements cell-to-cell movement of movement-deficient PVX mutant (PVXΔTGBp1-GFP) [23]. We investigated whether MP_FMV_ mutants (Trun, SP_spo_Trun and SP_cal_Trun) facilitate virus cell-to-cell movement using this system. SP_clv_Trun was not used in this experiment because its transient expression for more than 3 days caused cell death. The fluorescence of PVXΔTGBp1-GFP spread to adjacent cells when MP_FMV_ or SP_spo_Trun was co-expressed (Fig 5A), indicating that MP_FMV_ and SP_spo_Trun complemented cell-to-cell movement of PVXΔTGBp1-GFP. Quantitative analysis of fluorescence area suggested significant differences between MP_FMV_ or SP_spo_Trun and β-glucuronidase (GUS) control (Fig 5B). Categorizing the fluorescence area by the cell number per fluorescent spot also showed significant difference between MP_FMV_ or SP_spo_Trun and GUS control (p < 0.01 by Fisher's exact test). In contrast, the fluorescence of PVXΔTGBp1-GFP was almost confined to a single cell when co-expressed with Trun or SP_cal_Trun like GUS control (Fig 5A). Comparable expression levels of MP_FMV_ and its mutants were validated by Western blot analysis (Fig 5C). Thus, SP_spo_Trun, an SP chimera which has the ability to reach PD, complements cell-to-cell movement of PVXΔTGBp1-GFP, indicating that PD localization of MP_FMV_ is necessary to facilitate virus cell-to-cell movement.

**Fig 6:**
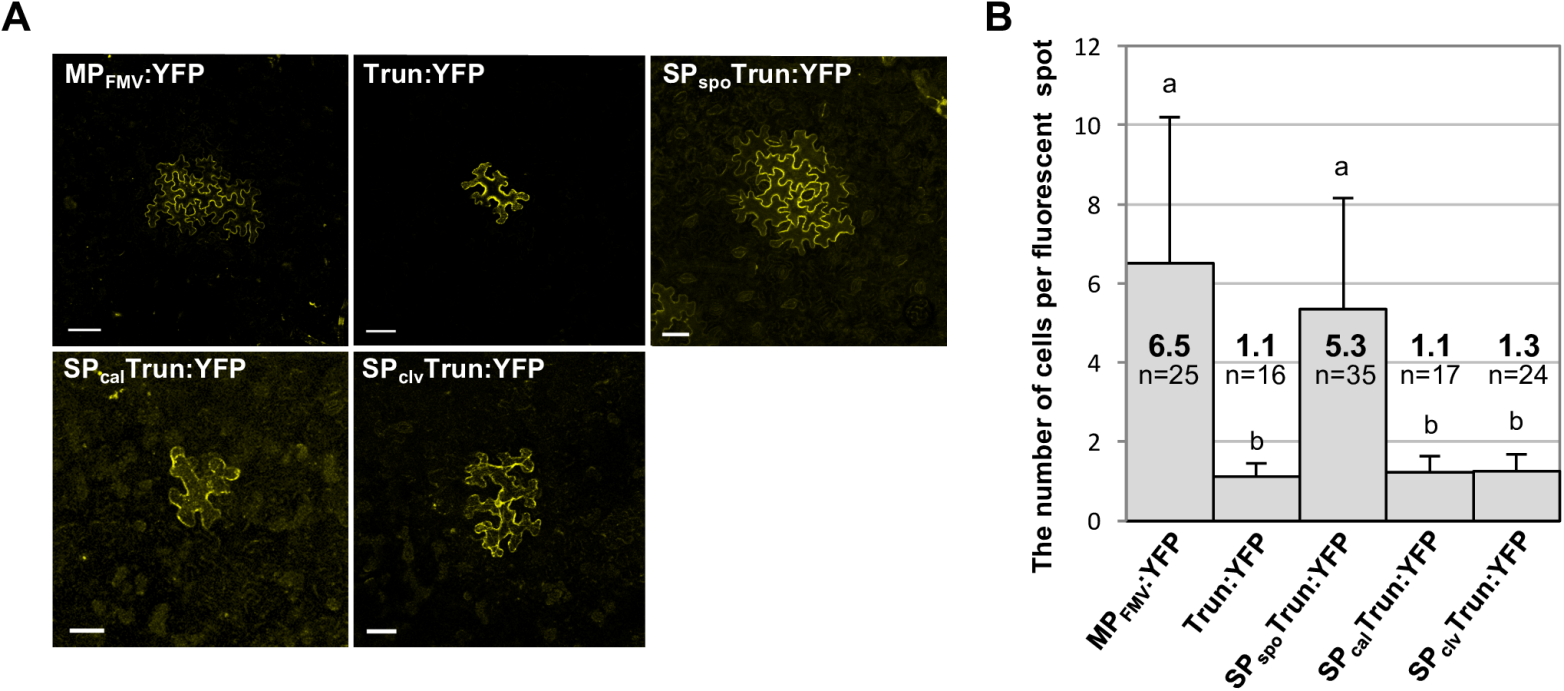
PD localization of MP_FMV_ is necessary for their cell-to-cell movement. Investigation of the ability of MP_FMV_ and its mutants (MP_FMV_:YFP, Trun:YFP, SP_spo_Trun:YFP, SP_cal_Trun:YFP and SP_clv_Trun:YFP) to move to adjacent cells. (A) Typical images were captured at 3 dpi. Bars = 50 μm. (B) Quantitative analysis. The bars show means + SD. n indicates the total number of measurements in two independent experiments. Different letters on error bars indicate statistical differences at the 1% level of significance (Tukey-Kramer test).

Most virus MPs are known to move to adjacent cells autonomously [30]. We assessed whether or not these MP_FMV_ mutants were able to move to adjacent cells. MP_FMV_:YFP and SP_spo_Trun:YFP spread to adjacent cells when expressed in a single cell (Fig 6A). Conversely, Trun:YFP, SP_cal_Trun:YFP and SP_clv_Trun:YFP, which do not have the PD-targeting ability, did not move to adjacent cells. Quantitative analysis suggested a significant difference between these two groups in the ability to move to adjacent cells (Fig 6B). These results are in good accordance with virus cell-to-cell complementation assay (Fig 5).

### SP-deficient MP_FMV_ localizes to PD when co-expressed with MP_FMV_ or SP chimeras

The experiments described above suggested that the translocation efficiencies determine the subcellular distribution and function of MP_FMV_. SP_cal_Trun and SP_clv_Trun, whose SPs have high translocation efficiencies, localized to the ER, whereas Trun, which does not have an SP, localized to the PM (Table 1). SP_spo_Trun, which have moderate translocation efficiency, were able to localize to PD in addition to the ER and the PM. Given that SP_FMV_ has low translocation efficiency, a small fraction of MP_FMV_ was probably recruited to the ER. These results allowed us to speculate that co-existence of ER-translocated and non-translocated MP_FMV_ is required for PD localization; in other words, ER-translocated MP_FMV_ and microdomain-localized MP_FMV_ act cooperatively to reach PD.

**Table 1:** Properties of MP_FMV_ and its mutants.

mutant	translocation efficiency (%)	Localization			cell-to-cell movement (cells)	complementation of virus movement (relative unit)
Trun	no (0)		PM		1.1 ± 0.3	1.7 ± 0.8
MP_FMV_ (wt)	low (4.3 ± 1.5)	(ER)	PM	PD	6.5 ± 3.6	5.6 ± 3.8
SP_spo_Trun	medium (70.9 ± 0.9)	ER	PM	PD	5.3 ± 2.8	4.2 ± 3.2
SP_cal_Trun	high (91.9 ± 3.9)	ER			1.1 ± 0.3	2.5 ± 1.4
SP_clv_Trun	high (93.0 ± 1.6)	ER			1.3 ± 0.4	ND

**Fig 7:**
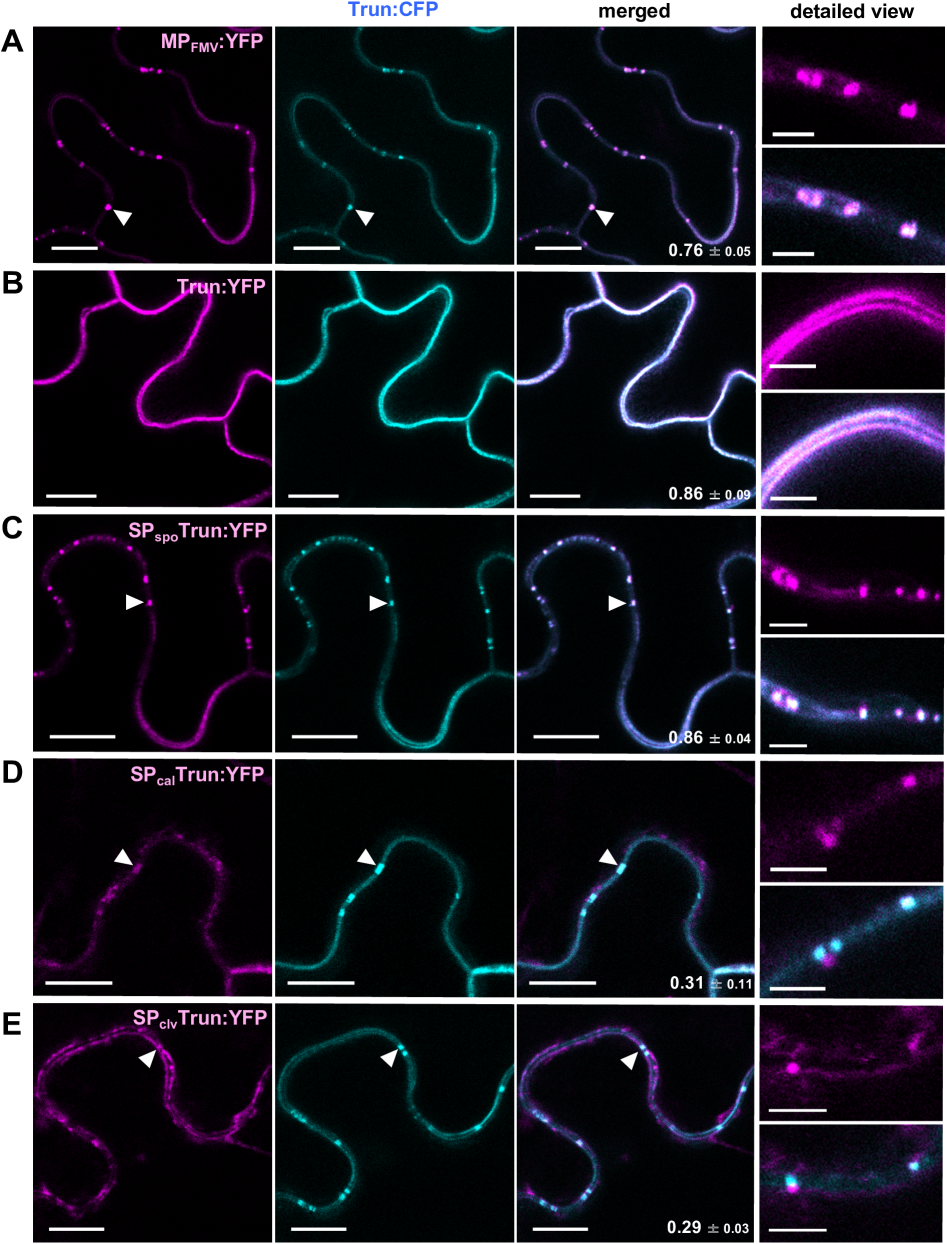
SP-deficient MP_FMV_ localizes to PD when co-expressed with MP_FMV_ or SP chimeras. Trun:CFP was co-expressed with (A) MP_FMV_:YFP, (B) Trun:YFP, (C) SP_spo_Trun:YFP, (D) SP_cal_Trun:YFP or (E) SP_clv_Trun:YFP. Cells were observed at 36 hpi. YFP fluorescence was pseudocolored with magenta. Arrowheads indicate localization to PD. Bars in images of detailed view are 2.5 μm. The others are 10 μm.

We tested this hypothesis by co-expressing SP-deficient MP_FMV_ fused with CFP (Trun:CFP) and MP_FMV_:YFP, Trun:YFP, SP_spo_Trun:YFP, SP_cal_Trun:YFP or SP_clv_Trun:YFP. Localization pattern was not altered when Trun:YFP and Trun:CFP were co-expressed (Fig 7B). By contrast, Trun:CFP was found to localize to punctate structures along the PM when co-expressed with MP_FMV_:YFP, SP_spo_Trun:YFP, SP_cal_Trun:YFP and SP_clv_Trun:YFP (Fig 7A and 7C–7E). Aniline blue staining confirmed that these punctate structures formed by Trun when co-expressed with MP_FMV_ and SP chimeras coincided with PD (PCC: 0.50 ± 0.06, 0.60 ± 0.06, 0.61 ± 0.05 and 0.56 ± 0.15, respectively; S4 Fig). YFP-fused MP_FMV_ and SP chimeras did not alter their localization by the expression of Trun:CFP. Detailed views showed that SP_cal_Trun:YFP and SP_clv_Trun:YFP localized in close proximity to, but did not substantially colocalize with Trun:CFP (Fig 7D and 7E; PCC: 0.31 ± 0.11 and 0.29 ± 0.03, respectively). This localization pattern was similar to those observed when SP_cal_Trun:YFP or SP_clv_Trun:YFP was expressed alone (Fig 4Ciii and 4Diii). These results suggest that Trun, which normally localized to the PM, was transported to PD by the function of MP_FMV_ or SP chimeras, which were at least partially translocated to the ER.

The fact that MP_FMV_ or SP chimeras changed Trun localization raised the possibility of the physical interaction between microdomain-localized MP_FMV_ and ER-translocated MP_FMV_. We have now investigated the interaction between MP_FMV_ and Trun or SP_clv_Trun using bimolecular fluorescence complementation (BiFC). We first co-expressed the basic leucine zipper transcription factor bZIP63 fused with N-terminal half of YFP (bZIP63:NYF) and with C-terminal half of YFP (bZIP63:CYF) as a control [31]. In this combination, strong fluorescence was observed in the nuclei (S5 Fig). Next, we tested the interaction between MP_FMV_:NYF and MP_FMV_:CYF, Trun:CYF or SP_clv_Trun:CYF. Co-expression of MP_FMV_:NYF and MP_FMV_:CYF or Trun:CYF showed a weak fluorescence on the PM. This fluorescence can be ascribed to a background signal or a weak dimerization of microdomain-localized MP_FMV_. Co-expression of MP_FMV_:NYF and SP_clv_Trun:CYF showed no signal, and the physical interaction between microdomain-localized MP_FMV_ and ER-translocated MP_FMV_ was not suggested.

### ER-translocated MP_FMV_ specifically localizes to ER-PM contact sites

**Fig 8:**
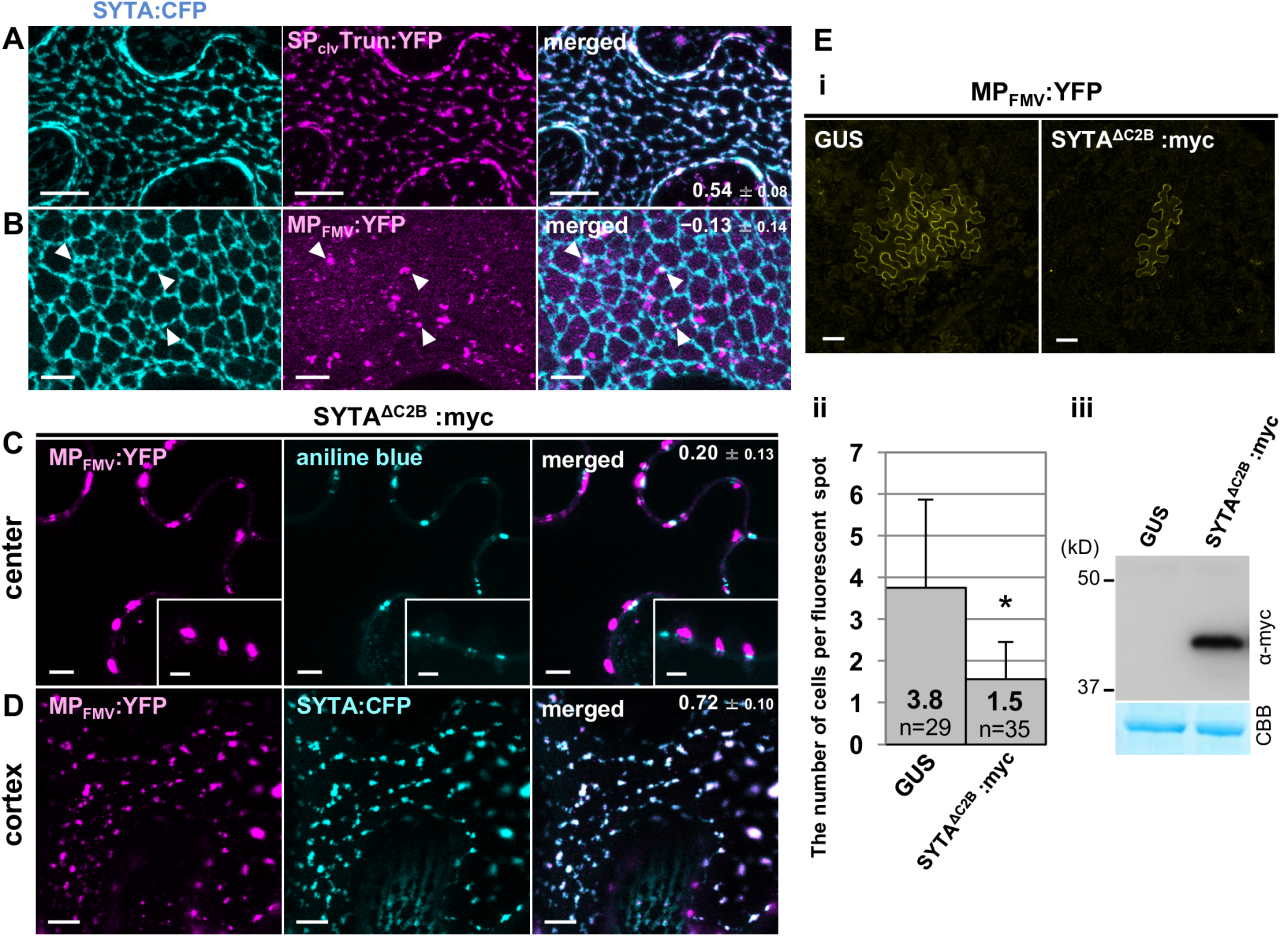
ER-translocated MP_FMV_ specifically localizes to ER-PM contact sites. (A and B) ER-translocated MP_FMV_ co-localized with SYTA, a protein localized to ER-PM contact sites. 3D-projection images of cells expressing SYTA:CFP and (A) SP_clv_Trun:YFP or (B) MP_FMV_:YFP. Z-section images of 10 slices at 1.0 μm intervals were processed. Arrowheads indicate co-localization of SYTA:CFP and MP_FMV_:YFP. (C and D) Expression of SYTA^ΔC2B^, a dominant-negative form of SYTA, affected MP_FMV_ localization. (C) SYTA^ΔC2B^:myc was co-expressed with MP_FMV_:YFP. Plasmodesmata were visualized by aniline blue treatment. (D) SYTA^ΔC2B^:myc was co-expressed with MP_FMV_:YFP and SYTA:CFP. A cortical region was visualized. (A–D) Cells were observed at 36 hpi. YFP fluorescence was pseudocolored with magenta. Bars: (A–D), 5 μm; (C) inset 2.5 μm. (E) SYTA^ΔC2B^:myc showed an inhibitory effect on MP_FMV_ movement to adjacent cells. MP_FMV_:YFP was co-expressed with GUS or SYTA^ΔC2B^:myc. (i) Typical images were captured at 3 dpi. Bars = 50 μm. (ii) Quantitative analysis. The bars show means + SD. n indicates the total number of total measurements in two independent experiments. The asterisk above an error bar indicates a statistical difference at the 1% level of significance (Student's t-test). (iii) Immunoblot analysis using anti-myc antibody (top panel). CBB staining is shown as a loading control (bottom panel). Samples were collected at 36 hpi.

Given that SP_cal_Trun:YFP and SP_clv_Trun:YFP localized in the close proximity to PD (Fig 4Ciii and 4Diii and Fig 7D and 7E), translocated MP_FMV_ appeared to localize in the specific region of the ER. We noticed that the distribution pattern of SP_cal_Trun:YFP and SP_clv_Trun:YFP was similar to that of the ER-PM contact site-associated protein, synaptotagmin1 (SYTA) [14,32], which is known to be involved in virus cell-to-cell movement [15–17]. Prior to the co-expression with MP_FMV_:YFP, we first analyzed localization of SYTA. Co-expression of SYTA:CFP and ER-YFP showed that SYTA:CFP was predominantly distributed to nodes of the ER (PCC = 0.61 ± 0.01; S6A Fig) consistent with the previous reports [14,15]. Aniline blue staining showed that SYTA:CFP was in close proximity to PD (PCC = 0.21 ± 0.01; S6B Fig), which was similar to those seen in SP_cal_Trun:YFP and SP_clv_Trun:YFP (Fig 7D and 7E). The close proximity of ER-PM contact sites and PD was reported also in a previous study [32]. To assess whether translocated MP_FMV_ localize to ER-PM contact sites, SYTA:CFP was co-expressed with SP_clv_Trun:YFP or MP_FMV_:YFP. SP_clv_Trun:YFP co-localized almost perfectly with SYTA:CFP, probably in the ER nodes (PCC = 0.54 ± 0.08; Fig 8A). A large fraction of MP_FMV_:YFP was distributed to the PM and PD, but a small fraction of punctate structures colocalized with SYTA:CFP (Fig 8B). These results show that ER-translocated MP_FMV_ specifically localized to ER-PM contact sites.

To obtain more information about the role of ER-PM contact sites in MP_FMV_ trafficking, we constructed a c-myc tagged SYTA^ΔC2B^ (SYTA^ΔC2B^:myc), which is a dominant-negative form lacking the C-terminal 177 aa of SYTA [16]. When MP_FMV_:YFP was expressed together with SYTA^ΔC2B^:myc, localization of MP_FMV_:YFP was apparently affected (Fig 8C and 8D). Although a small fraction of MP_FMV_:YFP was still retained in PD, a large proportion of MP_FMV_:YFP excessively accumulated next to PD (PCC = 0.20 ± 0.13; Fig 8C). MP_FMV_:YFP substantially colocalized with SYTA:CFP in the cortex; instead, fluorescence from MP_FMV_ that localized in the PM microdomains became weaker (PCC = 0.72 ± 0.10; Fig 8D). This result suggests that MP_FMV_:YFP that normally localized to the PM microdomains aberrantly accumulated in ER-PM contact sites by the expression of SYTA^ΔC2B^:myc.

We also investigated whether SYTA^ΔC2B^ affects cell-to-cell movement of MP_FMV_:YFP. Expression of SYTA^ΔC2B^:myc inhibited MP_FMV_:YFP movement to adjacent cells compared with when expressed with GUS (Fig 8Ei and 8Eii). Immunoblot analysis using anti-myc antibody confirmed the expression of SYTA^ΔC2B^:myc (Fig 8Eiii). Taken together, these data suggest that translocated MP_FMV_ localized to ER-PM contact sites and played an essential role in cell-to-cell movement. To see whether MP_FMV_ interacts with SYTA, BiFC was carried out. Fluorescent signal was not observed when MP_FMV_:NYF, Trun:NYF and SP_clv_Trun:NYF were co-expressed with SYTA:CYF (S7 Fig). Thus, the interaction between SYTA and MP_FMV_ or MP_FMV_ mutants was not suggested by BiFC.

### MP_FMV_ localization is not affected by inhibiting COPII transport

As the MPs of several viruses use the secretory pathway [9,33], involvement of COPII transport in MP_FMV_ trafficking has been verified by BFA treatment or expression of a dominant-negative form of Sar1 [Sar1(H74L)] [34]. We first confirmed that BFA treatment and Sar1(H74L) expression caused retention of the Golgi marker ManI:CFP [35] in the ER, as expected (S8 Fig). PD localization in cells expressing MP_FMV_:YFP was not affected by BFA treatment or Sar1(H74L) expression. Similarly, inhibiting COPII transport did not affect localization of Trun:YFP in the PM. These results suggest that COPII transport is not involved in the subcellular localization of MP_FMV_ and that MP_FMV_ uses pathways different from BFA-sensitive MPs [9,33].

## Discussion

**Fig 9:**
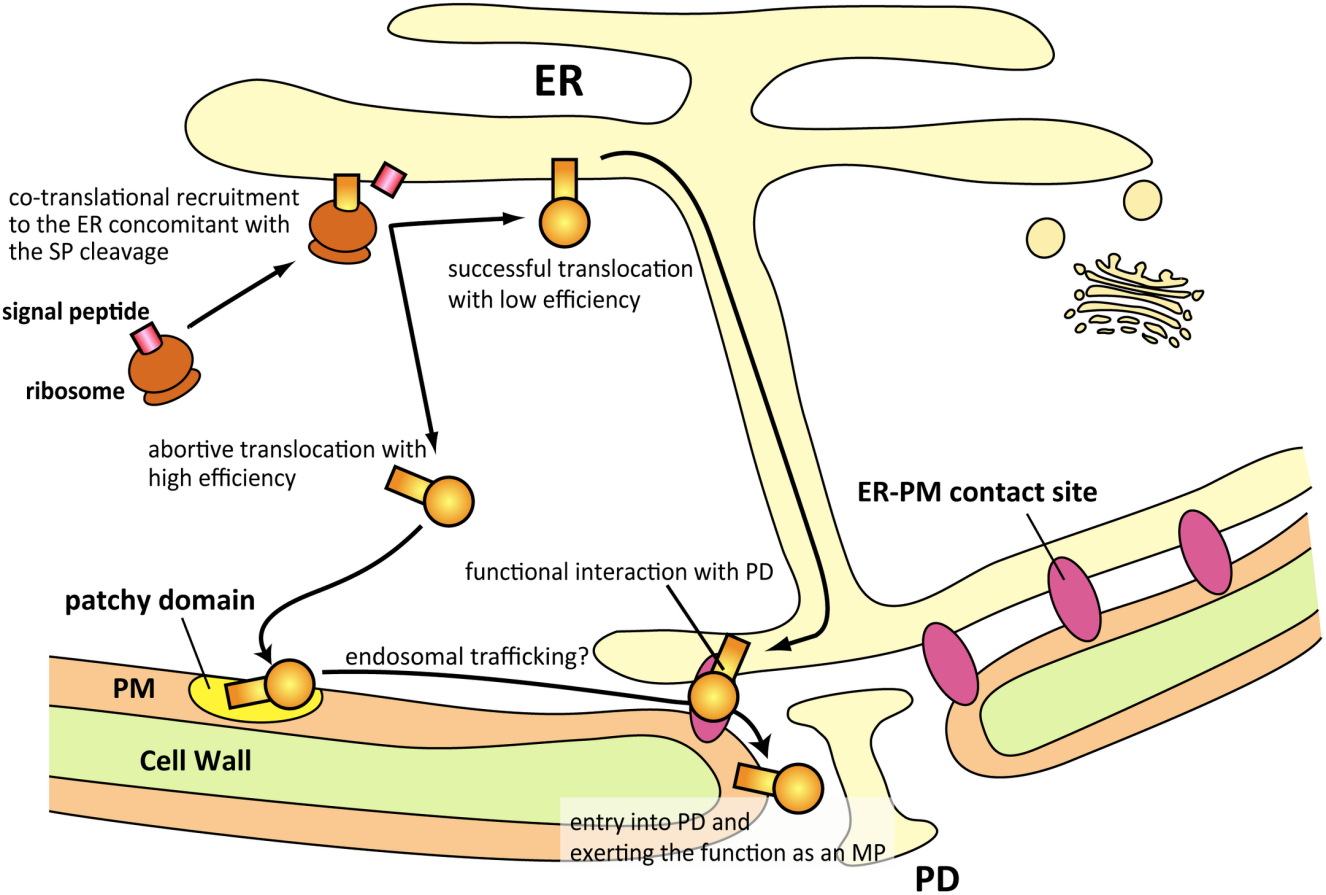
A model for the intracellular trafficking of MP_FMV_.

In this study, we analyzed the intracellular trafficking of MP_FMV_, focusing on SP function, and found that MP_FMV_ targets two subdomains in the ER and PM as well as PD. MP_FMV_, which has an N-terminal SP, was distributed mainly in PD and patchy microdomains of the PM (Fig 1 and Fig 2). Investigation of ER translocation efficiency revealed that SP_FMV_ has much lower translocation efficiency compared with those of SP_spo_, SP_cal_ and SP_clv_ (Fig 3). The SP-deficient mutant (Trun) exclusively localized to the PM microdomains (Fig 4A), whereas two SP chimeras (SP_cal_Trun and SP_clv_Trun) exclusively localized to the ER (Fig 4C and 4D). SP_spo_Trun was distributed in the ER, PM microdomains and PD similar to MP_FMV_, even though a portion of SP_spo_Trun aggregated in the ER (Fig 4B). The results so far indicated that MP_FMV_ dually targets the ER and PM due to the inefficient SP; a fraction of MP_FMV_ was successfully translocated into the ER, whereas the remainder of MP_FMV_, which failed to be translocated, is transferred to the microdomains. This finding led us to speculate that both ER-translocated MP_FMV_ and microdomain-localized MP_FMV_ are necessary for PD localization. Consistent with this notion, the SP-deficient mutant entered into PD by the expression of MP_FMV_ or the SP chimeras, which are able to, at least partially, translocate into the ER (Fig 7). Furthermore, we showed that translocated MP_FMV_ specifically localized to ER-PM contact sites (Fig 8A and 8B), and dominant-negative inhibition of SYTA affected PD localization and cell-to-cell movement of MP_FMV_ (Fig 8C–8E). These results suggest that MP_FMV_ localized to ER-PM contact sites plays an essential role in the entry of microdomain-localized MP_FMV_ into PD. PD localization of MP_FMV_ is necessary to facilitate cell-to-cell movement as shown in the virus movement complementation assay (Fig 5) and the cell-to-cell movement assay (Fig 6). Altogether, we propose a new model for the intracellular trafficking of a viral MP. A substantial proportion of MP_FMV_, which failed to be translocated, is directly transferred to the microdomains, whereas the remainder of MP_FMV_, which successfully translocated into the ER, subsequently localizes to ER-PM contact sites and functionally interact with PD to enable microdomain-localized MP_FMV_ to enter into PD (Fig 9).

Dual targeting of MP_FMV_ to the ER and the PM is explained by the low translocation efficiency of the SP_FMV_ (Fig 3). In general, proteins with an SP are co-translationally recognized by the signal recognition particle in the cytosol and recruited to the signal recognition particle receptor on the ER membrane. Then, the Sec61p complex, a channel integrated into the ER membrane, translocates these preproteins into the ER, and subsequently the SP is cleaved by a membrane-bound SP peptidase on the lumenal side [36]. Therefore, the finding that MP_FMV_ was translocated to the ER at a lower rate (approximately 5%; Fig 3) despite the cleavage of SP_FMV_ (Fig 2) is surprising. A substantial proportion of MP_FMV_ molecules probably abort ER translocation after SP cleavage and are released into the cytosol, but further studies are needed to reveal the detailed mechanism of the abortion. In animal cells, it has been reported that an inefficient SP of an ER chaperone calreticulin regulates the ratio of translocated and nontranslocated populations [37]. However, the inefficiency of the animal calreticulin SP is quite moderate, and it generates only a small nontranslocated population. In this regard, to our knowledge, this is the first report of an SP with extremely low efficiency that controls the subcellular distribution of the nascent protein. This low-efficiency SP_FMV_ generates only a small ER-translocated population, but it is probably sufficient for the entry of microdomain-localized MP_FMV_ into PD considering that SP_spo_Trun:YFP, whose SP has medium translocation efficiency, excessively accumulated in the ER compared with the case of MP_FMV_:YFP (Fig 4B). SP_FMV_ likely regulates the distribution of MP_FMV_ between the PM and ER in the appropriate proportion.

How is MP_FMV_ associated with two different types of the cellular membrane, the ER membrane and PM? Curiously, a transmembrane domain in MP_FMV_ has not been predicted by SOSUI (http://harrier.nagahama-i-bio.ac.jp/sosui/) and TMHMM server v. 2.0 (http://www.cbs.dtu.dk/services/TMHMM/). Localization of the MP_FMV_ to the PM microdomains is explained as a peripheral membrane protein. Given that the SP-deficient MP_FMV_ was exclusively associated with the PM microdomains (Fig 4A), even ER translocation is not required to localize to PM microdomains. Microdomain-associated proteins, remorins and flotillins, which do not have SPs and transmembrane domains similarly, are suggested to be peripherally associated with the PM [38–40]. In other words, a transmembrane domain and penetrating the membrane are dispensable for the localization to PM microdomains. The association with the ER membrane is explained by the abnormality of viral proteins. Recent studies on the topology of viral MPs revealed that MPs of tobacco mosaic virus and tomato spotted wilt virus are suggested to be associated with the ER membrane using unusual hydrophobic regions [41–43]. MP_FMV_ may also establish such unconventional hydrophobic regions to be associated with the ER membrane.

Our results raise the question of how two populations of MP_FMV_ that localize to the ER and PM subdomains act cooperatively to gain access to PD. According to BiFC analysis (S7 Fig), physical interaction of these two population is not likely. Although only limited information is available on these membrane subdomains, one possible explanation is that MP_FMV_ in ER-PM contact sites functionally interact with PD, and this might allow the microdomain-localized MP_FMV_ to access into PD (Fig 9). This hypothesis is corroborated by the facts that ER-PM contact sites are spatially close to PD (S6B Fig) [32] and that expression of SYTA^ΔC2B^, a dominant-negative form of SYTA, affected PD and PM localization of MP_FMV_ (Fig 8C and 8D). In accord with our results, a previous study showed that, although the localization of 30K was not affected, the cell-to-cell movement of 30K was suppressed in an *Arabidopsis syta* mutant [16]. This study also showed that SYTA^ΔC2B^ inhibited the formation of endosomes, suggesting that SYTA regulates endocytosis [16]. From these facts, we suspect that MP_FMV_ in the patchy microdomains is transported into PD through endosomal trafficking regulated by SYTA (Fig 9).

The intimate relationship between PD and patchy domains in the PM is implied in this and in other studies. In our study, relocation of the microdomain-localized MP_FMV_ to PD (Fig 7) indicates a functional connection between the patchy microdomains and PD. One previous study about TMV 30K reported that dominant-negative inhibition of class VIII myosins affected PD localization of 30K and induced a patchy distribution in the PM, which did not merge with that of REM1.3 similar to the results from this study [44]. Originally, PM passing through PD is also recognized as a type of microdomain, as it is functionally and spatially distinguished from the surrounding PM [2,45]. Taken together, these two types of PM microdomains, the patchy domains and PM passing through PD, might be functionally connected by ER-PM contact sites.

This study also presents a functional differentiation of MP_FMV_ between the two populations, MP_FMV_ in ER-PM contact sites and PM microdomains. The functional differentiation of a virus MP is reminiscent of the mechanism of cell-to-cell movement regulated by more than one protein. For example, triple gene block movement proteins, which are encoded by viruses belonging to the *Virgaviridae*, *Alphaflexiviridae* and *Betaflexiviridae* families, have specialized functions and perform different tasks for virus cell-to-cell movement: delivering viral factors to PD, interacting with host factors, and increasing PD permeability [46,47]. MP_FMV_ plays multiple roles in cell-to-cell movement using the SP_FMV_ with low translocation efficiency, probably to avoid splitting into modules. This concept may be true also in plant proteins. Although a number of studies have shown that a diverse array of plant proteins have N-terminal SPs, their ER translocation abilities have seldom been investigated. Our findings raise the possibility that SPs can potentially regulate subcellular distribution of the nascent proteins and contribute to protein function in plant cells.

## Materials and methods

### Transient protein expression

Plasmids expressing GUS, MP_FMV_, MP_FMV_:YFP and MP_FMV_:CFP were prepared as described earlier [23]. Expression vectors of ER-CFP and ER-YFP were purchased from the Arabidopsis Biological Resource Center (Stock numbers CD3-953 and CD3-957, respectively). MP_FMV_ mutant whose SP is not cleaved (ncMP_FMV_) was generated by an PCR using primers containing substitutions to introduce L7P and V11P mutations. ncMP_FMV_ was cloned into pEarleyGate 101 (ncMP_FMV_:YFP) using Gateway technology [48,49]. Trun sequence, which lacks the N-terminal 19 aa of MP_FMV_, was amplified by PCR and cloned into pEarleyGate 100 (Trun), pEarleyGate 101 (Trun:YFP) and pEarleyGate 102 (Trun:CFP). SP chimera sequences were amplified by PCRs using primers containing each SP sequence, followed by cloning into pEarleyGate 100 (SP_spo_Trun, SP_cal_Trun and SP_clv_Trun) or 101 (SP_spo_Trun:YFP, SP_cal_Trun:YFP and SP_clv_Trun:YFP). In these SP chimeras, the SP region of MP_FMV_ was replaced with the N-terminal sequences of *Ipomoea batatas* sporamin A (M16861; 24 aa) [26], *Nicotiana tabacum* calreticulin (EU984501; 28 aa) [27] or *A*. *thaliana* CLAVATA3 (AF126009; 22 aa) [28], each of which contains an SP sequence and one aa downstream of the cleavage site. MP_FMV_:FLAG, in which MP_FMV_ was fused to a FLAG epitope tag immediately downstream of its C terminus, was amplified by PCRs using primers containing FLAG sequence, and cloned into pEarleyGate 100. A 3× N-glycosylation sequon [29] or an ER-retention signal [50] were introduced to the GFP sequence in their C terminus (GFPglc and GFP:HDEL) as was the case with MP_FMV_:FLAG. The GFP sequence was derived from pEarleyGate 103. The SPs of MP_FMV_, sporamin A, calreticulin or CLAVATA3 were N-terminally added to GFPglc (SP_FMV_GFPglc, SP_spo_GFPglc, SP_cal_GFPglc and SP_clv_GFPglc) or GFP:HDEL (SP_FMV_GFP:HDEL, SP_spo_GFP:HDEL, SP_cal_GFP:HDEL and SP_clv_GFP:HDEL) as was the case with SP chimeras. The REM1.3 (At4g36970) and SYTA (At2g20990) sequences were amplified by PCR from total DNA of *A*. *thaliana*. *REM1*.*3* was cloned into pEarleyGate 104 (YFP:REM1.3), and *SYTA* was cloned into pEarleyGate 101 (SYTA:YFP) and pEarleyGate 102 (SYTA:CFP). *SYTA*^*ΔC2B*^, a dominant-negative form of *SYTA*, was amplified by PCR according to a previous study [16], and cloned into pEarleyGate Cmyc (SYTA^ΔC2B^:myc). pEarleyGate Cmyc is an in-house expression vector built from pEarleyGate 101 to introduce a myc tag at the C terminus of a cloned gene. Vectors for BiFC analysis were constructed as shown in the previous study [31]. The TMV MP:GFP sequence (30K:GFP) was amplified from pTMV-MP:GFP [51], and cloned into the pBI121 vector using SalI and BamHI sites.

### Plant materials and transient expression

The upper leaves of four-week-old *N*. *benthamiana* plants were used for the transient expression assays. Transient expression was mediated by infiltration of *Agrobacterium tumefaciens* as described previously [23].

### Confocal imaging

Cells expressing fluorescent protein fusions were imaged using a Leica TCS SP5 laser-scanning confocal microscope. An HCX PL Apo 63×/1.4–0.6 oil CS lens was used for imaging subcellular localization of fluorescent protein fusions and an HC PL Apo 10×/0.4 CS lens was used for imaging cell-to-cell movement of fluorescent protein fusions. Cells expressing CFP and/or YFP fusions were visualized as described earlier [52]. GFP was excited at the 488-nm argon laser line, and the emission was visualized at 500 to 600 nm. For PM staining, leaves were infiltrated with 50 μM FM4-64 in distilled water, and observed at 1 hpi. FM4-64 was excited at the 543-nm helium/neon laser line, and the emission was visualized at 580 to 650 nm. For PD staining, leaves were infiltrated with 0.1% (w/v) aniline blue in 50 mM sodium phosphate buffer (pH 9.0), and cells were observed at 2 hpi. Aniline blue was excited at the 405-nm laser line, and the emission was visualized at 425 to 480 nm. All the images were acquired at room temperature. Confocal images were processed with LAS AF software version 2.7.3 and Adobe Photoshop CS4. For deconvolution image analysis, between 3 and 8 z-section images at 0.15 μm intervals were captured and processed with Leica Hyvolution system. Fluorescence intensity graphs were generated using the LAS AF quantify intensity tool. PCCs were measured using an Fiji Colocalization plugin [53], and the mean values and standard deviations were calculated from three different images. Generally, PCC values of 0.2–0.4 indicate weak positive correlations and PCC values above 0.5 indicate strong positive correlations [54].

### Plasmolysis and inhibition assay

Leaves expressing fluorescent protein fusions were immersed in 4% (w/v) NaCl for 15 min for plasmolysis. For the inhibition assay, leaves were treated with 50 μg/ml BFA in 0.5% (v/v) dimethyl sulfoxide at 18 hpi and observed at 6 h after the treatment.

### Quantitative analysis of cell-to-cell movement

A GFP-tagged movement-defective mutant of the PVX infectious clone (PVXΔTGBp1-GFP) [55] was used in the virus movement complementation experiment. Two *Agrobacterium* cultures harboring the binary plasmid expressing GUS, MP_FMV_ or an MP_FMV_ mutant and PVXΔTGBp1-GFP were resuspended and mixed to final concentrations of OD_600_ = 0.4 and 0.0002, respectively. Leaves at 5 dpi were observed under an M165 FC fluorescence stereomicroscope (Leica Microsystems) with an ET GFP filter. Images were captured by a Leica DFC 310 FX camera and LAS software version 4.4.0. The areas of fluorescent foci were measured using ImageJ software version 1.40 (National Institutes of Health). In the assessment of cell-to-cell movement, an *Agrobacterium* culture harboring the binary plasmid expressing MP_FMV_ or its mutants was resuspended and diluted to OD_600_ = 0.0002. When cell-to-cell movement under condition of dominant-negative inhibition of SYTA was investigated, *Agrobacterium* cultures harboring the binary plasmid expressing SYTA^ΔC2B^:myc or GUS and MP_FMV_:YFP were resuspended and mixed to final concentrations of OD_600_ = 1.0 and 0.0002, respectively. Leaves at 3 dpi were observed under the laser-scanning confocal microscope as described above.

### Detergent treatment of membrane

FMV-infected fig leaves and *N*. *benthamiana* leaves transiently expressing MP_FMV_:FLAG or YFP:REM1.3 at 36 hpi were used for isolation of membrane-rich fraction (P30). The fractionation of P30 and chemical treatment were carried out according to the methods of Schaad et al. [56] with minor modifications as follows: Complete Mini (Roche Diagnostics) was added to buffer Q (Lysis Buffer) as a protease inhibitor instead of leupeptin, aprotinin and phenylmethylsulfonyl fluoride. P30 pellets were treated with buffer Q or 1% Triton X-100 (1%[v/v] TritonX-100, 25 mM Tris-HCl [pH 7.5], 150 mM NaCl and 5 mM EDTA).

### Immunoprecipitation and immunoblotting

MP_FMV_:FLAG was immunoprecipitated from cell lysate of *N*. *benthamiana* leaves expressing MP_FMV_:FLAG with EZview red anti-FLAG M2 affinity gel (Sigma-Aldrich) [52]. Immunoblot analysis was performed [52] using anti-FLAG M2 antibody (Sigma-Aldrich), anti-myc antibody (EMD Millipore), anti-Bip antibody (Santa Cruz Biotechnology, Inc.), anti-H^+^ATPase antibody (Agrisera) or anti-MP_FMV_ antibody. Anti-MP_FMV_ antibody, a polyclonal antibody against the mature region of MP_FMV_, was generated as described previously [52].

### Prediction of signal peptides

SignalP 4.1 software was used to predict SPs [57]. The accession number of MP_FMV_ sequence is BAM13816.

### Investigation of ER translocation efficiency

Deglycosylation using endoglycosidase H (Endo H; New England Biolabs) was carried out according to the manufacturer's instructions. Leaves transiently expressing GFPglc, SP_FMV_GFPglc, SP_spo_GFPglc, SP_cal_GFPglc or SP_clv_GFPglc at 30 hpi were homogenized in 1× glycoprotein denaturing buffer (0.5% SDS and 40 mM DTT), and incubated at 65°C for 15 min. After removal of cellular debris by centrifugation, the supernatant was suspended in 1× GlycoBuffer 3 (50 mM sodium acetate, pH 6.0) followed by the addition of distilled water or Endo H. After incubation at 37°C for 1 h, Endo H was inactivated at 75°C for 10 min. These samples were analyzed by immunoblotting using anti-GFP antibody (Roche). Signal intensity of each band was quantified using ImageJ software.

## Supporting information

S1 Fig Peptide sequence analysis of the MP_FMV_:FLAG N terminus. The result of Edman degradation. Letters in the chart indicate peaks corresponding to each amino acid. The N-terminal MP_FMV_ sequence is given below the chart.
(TIF)

S2 Fig Alignment of SP sequences used in this study. Purple boxes indicate conserved amino acid residues.
(TIF)

S3 Fig Localization of an MP_FMV_ mutant whose SP is not cleaved. (A) SP prediction of an MP_FMV_ mutant to which L7P and V11P substitutions are introduced (ncMP_FMV_). (B) Localization of ncMP_FMV_:YFP (pseudocolored magenta). (i) A bright-field image was merged. Dotted lines indicate the cell wall (CW). (ii) PD were stained with aniline blue. Cells were observed at 36 hpi. Bars = 5 μm.
(TIF)

S4 Fig Co-expression of SP-deficient MP_FMV_ and SP chimeras in aniline blue-stained cells. Trun:YFP (pseudocolored magenta) was co-expressed with MP_FMV_, Trun, SP_spo_Trun, SP_cal_Trun or SP_clv_Trun. Cells were stained with aniline blue and observed at 36 hpi. Bars = 10 μm.
(TIF)

S5 Fig Bimolecular fluorescence complementation (BiFC) to investigate the interaction between MP_FMV_ and MP_FMV_ mutants. MP_FMV_:NYF and MP_FMV_:CYF, Trun:CYF or SPclvTrun:CYF were co-expressed. bZIP63 was used as a control. Cells were observed at 36 hpi.
(TIF)

S6 Fig Localization analysis of SYTA. (A) 3D-projection images of cells expressing SYTA:CFP and the ER marker ER-YFP (pseudocolored magenta). Z-section images of 10 slices at 1.0 μm intervals were processed. (B) Aniline blue staining of SYTA:YFP-expressing cells. Cells were observed at 36 hpi. YFP fluorescence was pseudocolored with magenta. Bars: (A), 5 μm; (B) 10 μm; (B) inset 2.5 μm.
(TIF)

S7 Fig Bimolecular fluorescence complementation (BiFC) to investigate the interaction between MP_FMV_ or MP_FMV_ mutants and SYTA. MP_FMV_:NYF, Trun:NYF and SPclvTrun:NYF were co-expressed with SYTA:CYF. Cells were observed at 36 hpi. Bars = 25 μm.
(TIF)

S8 Fig PD localization is not affected by inhibitions of COPII transport. Whether COPII transport is involved in the localization of MP_FMV_:YFP and Trun:YFP was tested by treatments with 0.5%(v/v) dimethyl sulfoxide (DMSO), 50 μg/ml brefeldin A (BFA) or expression of Sar1(H74L). A Golgi marker, ManI:CFP was used as a control. Cells were observed at 24 hpi. Bars = 10 μm.
(TIF)
