# Supplementary figures and images for: Dual targeting of a virus movement protein to ER and plasma membrane subdomains is essential for plasmodesmata localization
Source: PLoS Pathog. 2017 Jun 22;13(6):e1006463. doi: 10.1371/journal.ppat.1006463 (PMC5498070; doi:10.1371/journal.ppat.1006463)

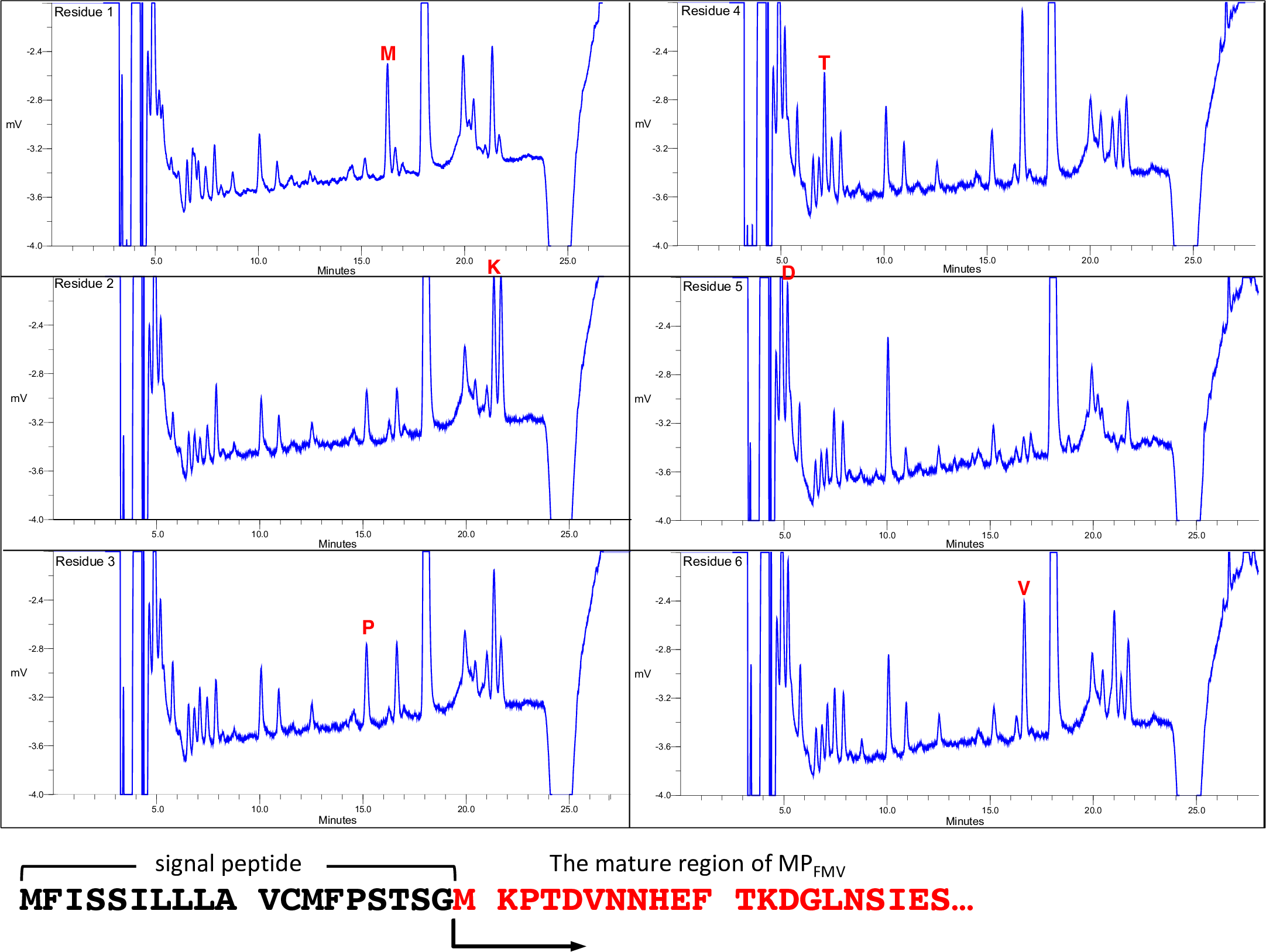

Supplement: S1 Fig — The result of Edman degradation. Letters in the chart indicate peaks corresponding to each amino acid. The N-terminal MPFMV sequence is given below the chart. (TIF) [file ppat.1006463.s001.tif]

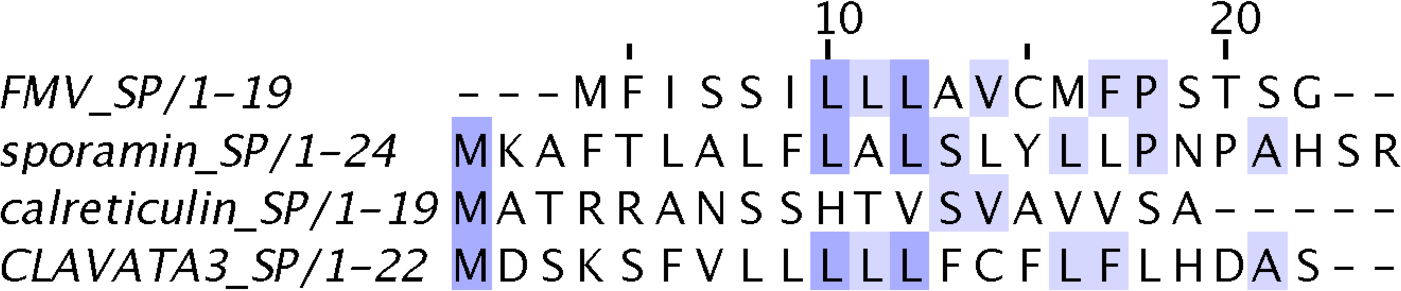

Supplement: S2 Fig — Purple boxes indicate conserved amino acid residues. (TIF) [file ppat.1006463.s002.tif]

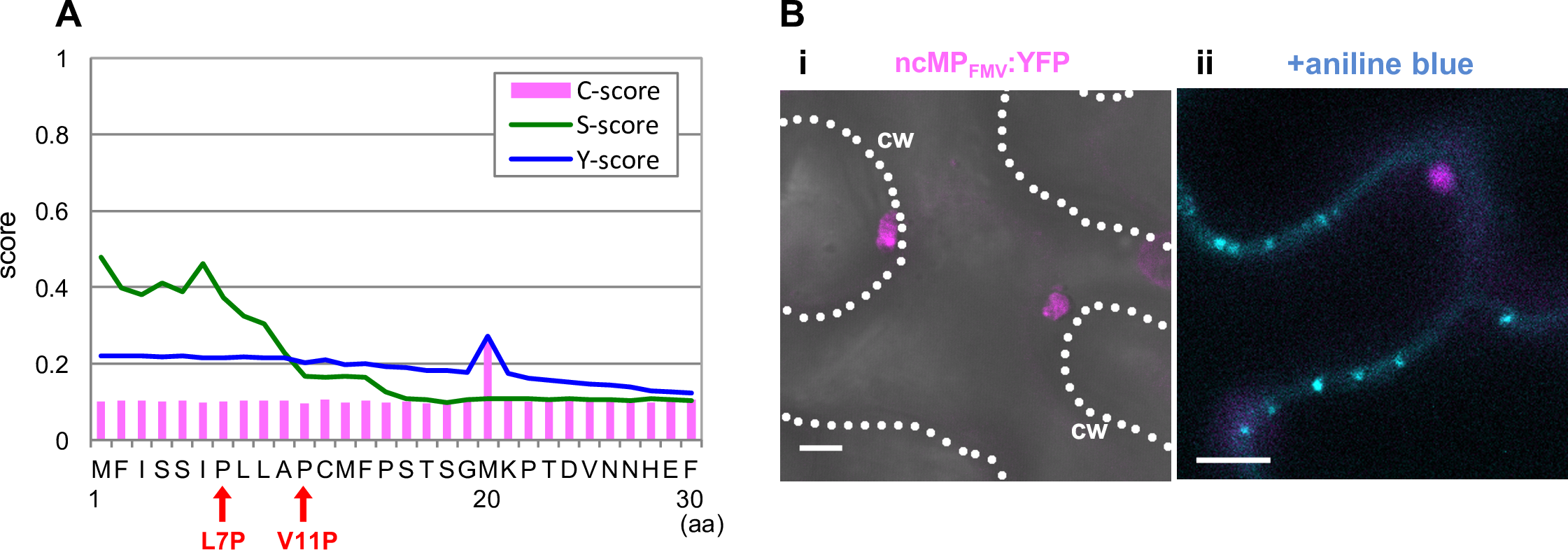

Supplement: S3 Fig — (A) SP prediction of an MPFMV mutant to which L7P and V11P substitutions are introduced (ncMPFMV). (B) Localization of ncMPFMV:YFP (pseudocolored magenta). (i) A bright-field image was merged. Dotted lines indicate the cell wall (CW). (ii) PD were stained with aniline blue. Cells were observed at 36 hpi. Bars = 5 μm. (TIF) [file ppat.1006463.s003.tif]

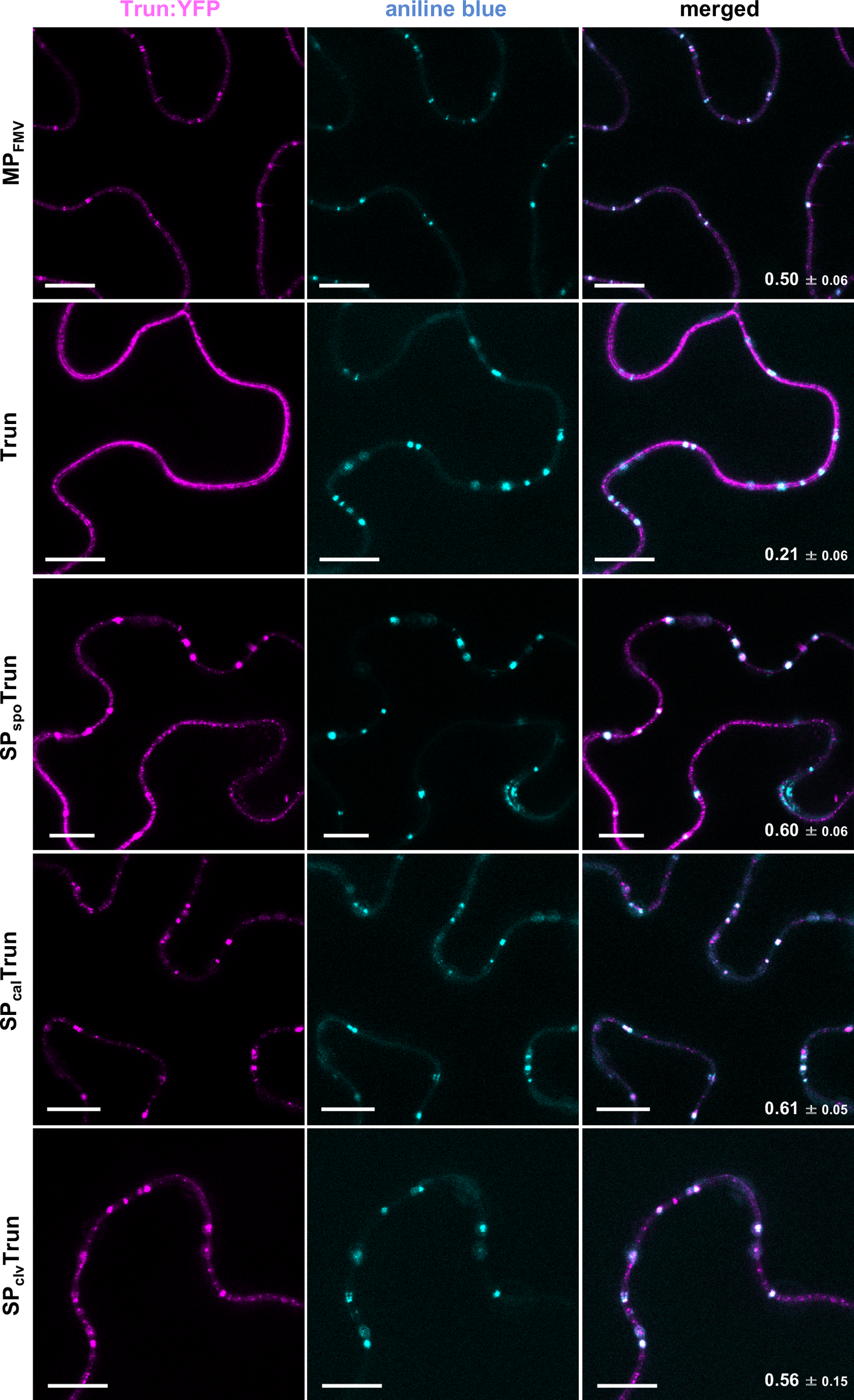

Supplement: S4 Fig — Trun:YFP (pseudocolored magenta) was co-expressed with MPFMV, Trun, SPspoTrun, SPcalTrun or SPclvTrun. Cells were stained with aniline blue and observed at 36 hpi. Bars = 10 μm. (TIF) [file ppat.1006463.s004.tif]

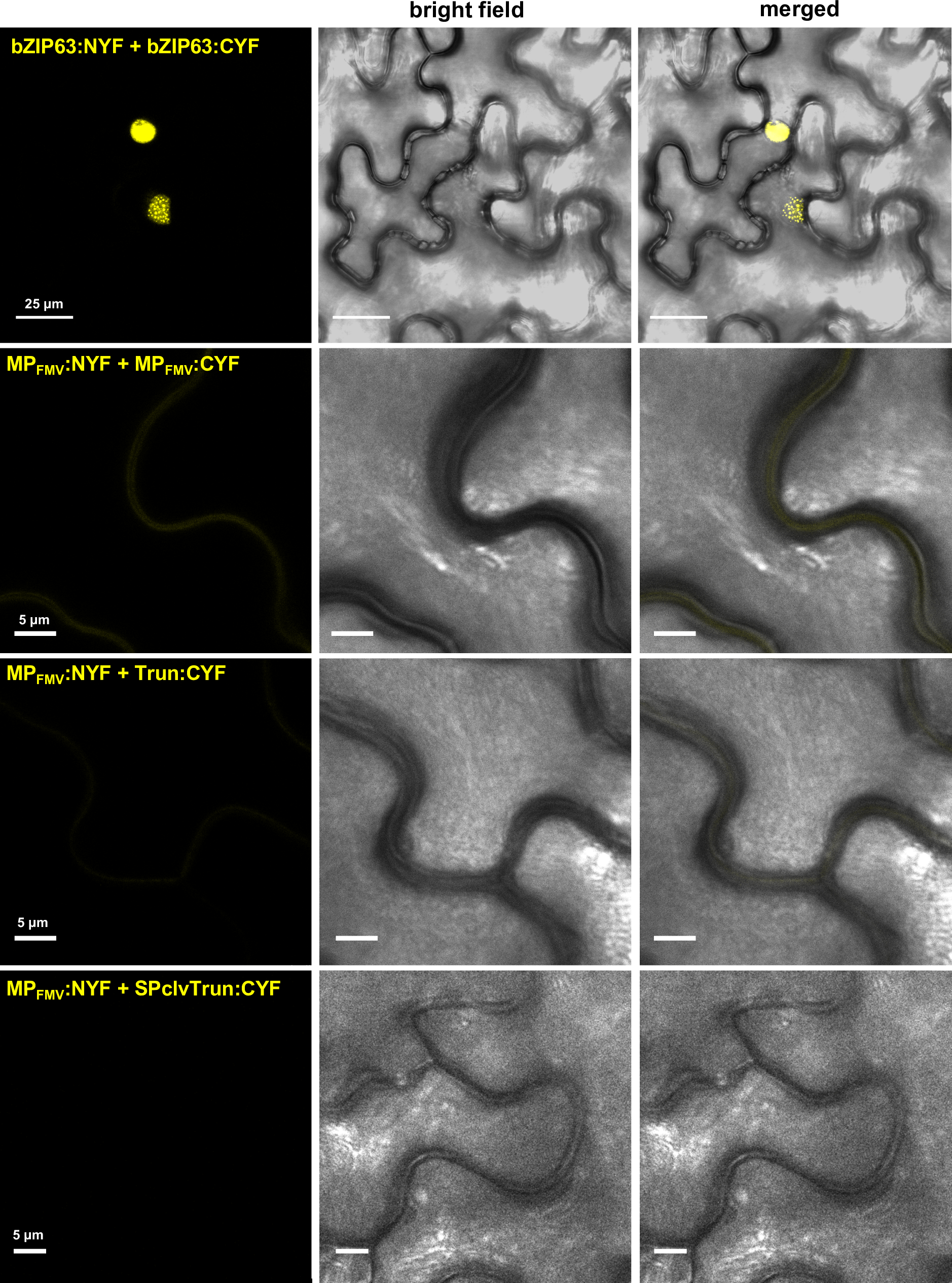

Supplement: S5 Fig — MPFMV:NYF and MPFMV:CYF, Trun:CYF or SPclvTrun:CYF were co-expressed. bZIP63 was used as a control. Cells were observed at 36 hpi. (TIF) [file ppat.1006463.s005.tif]

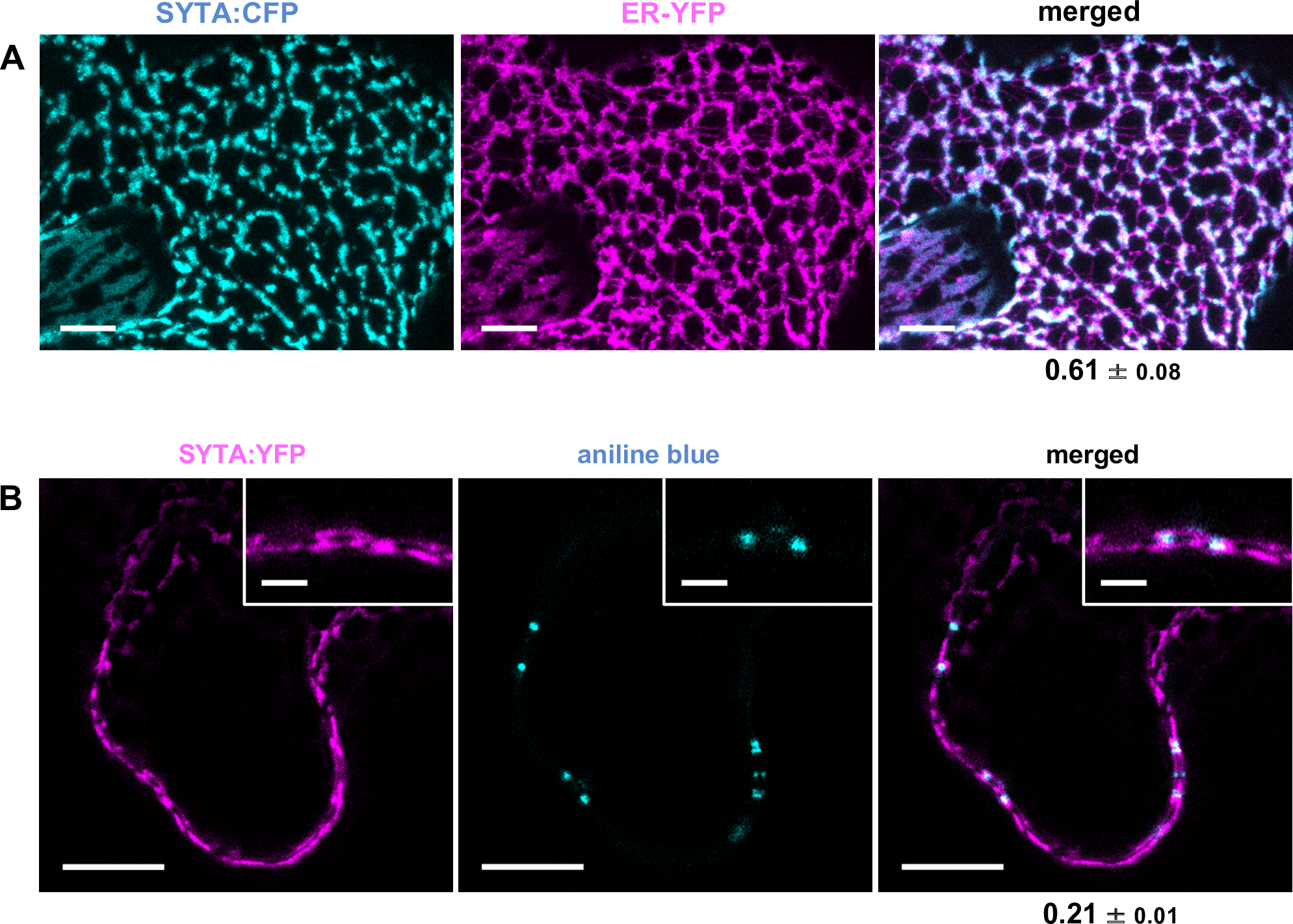

Supplement: S6 Fig — (A) 3D-projection images of cells expressing SYTA:CFP and the ER marker ER-YFP (pseudocolored magenta). Z-section images of 10 slices at 1.0 μm intervals were processed. (B) Aniline blue staining of SYTA:YFP-expressing cells. Cells were observed at 36 hpi. YFP fluorescence was pseudocolored with magenta. Bars: (A), 5 μm; (B) 10 μm; (B) inset 2.5 μm. (TIF) [file ppat.1006463.s006.tif]

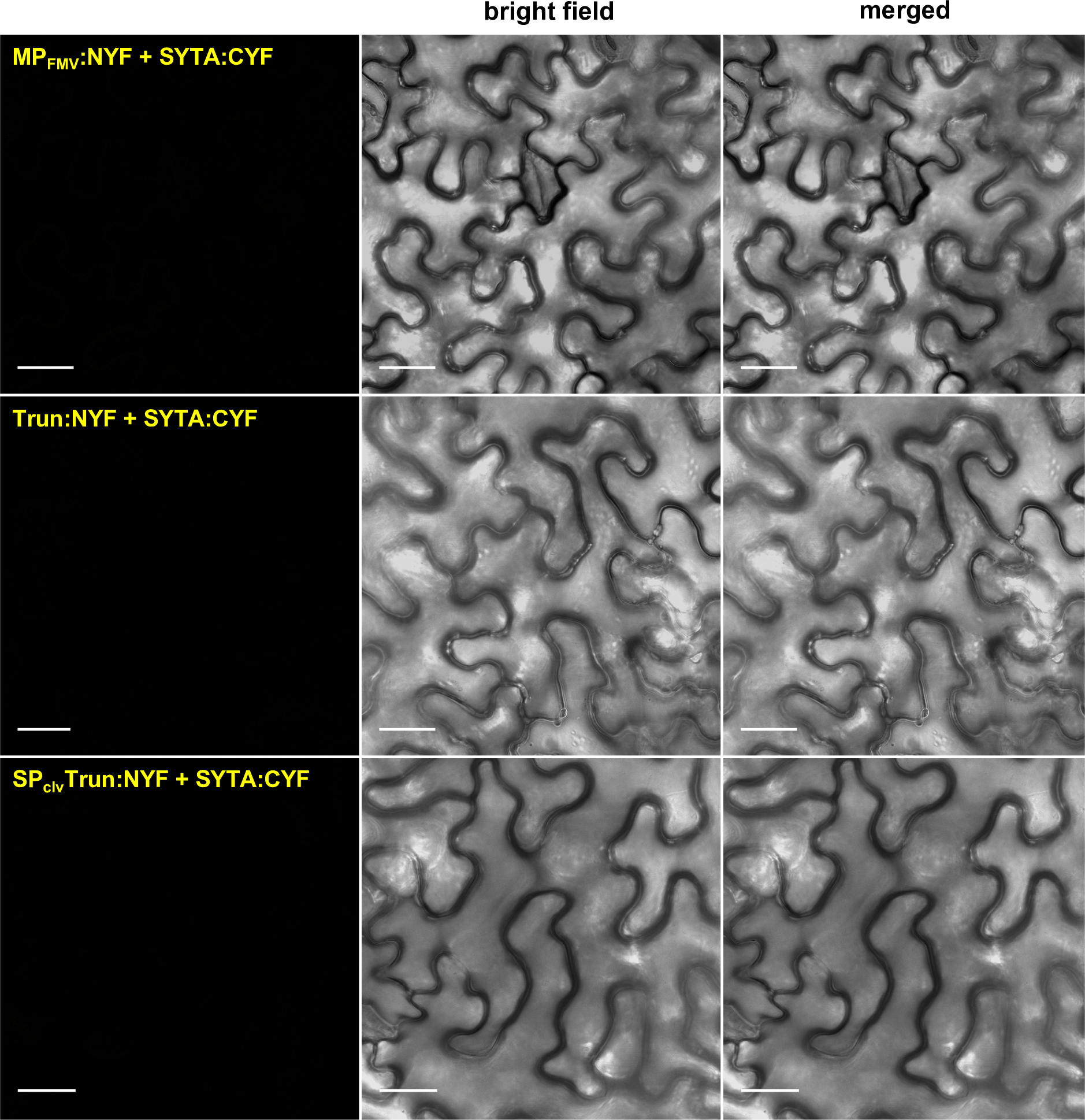

Supplement: S7 Fig — MPFMV:NYF, Trun:NYF and SPclvTrun:NYF were co-expressed with SYTA:CYF. Cells were observed at 36 hpi. Bars = 25 μm. (TIF) [file ppat.1006463.s007.tif]

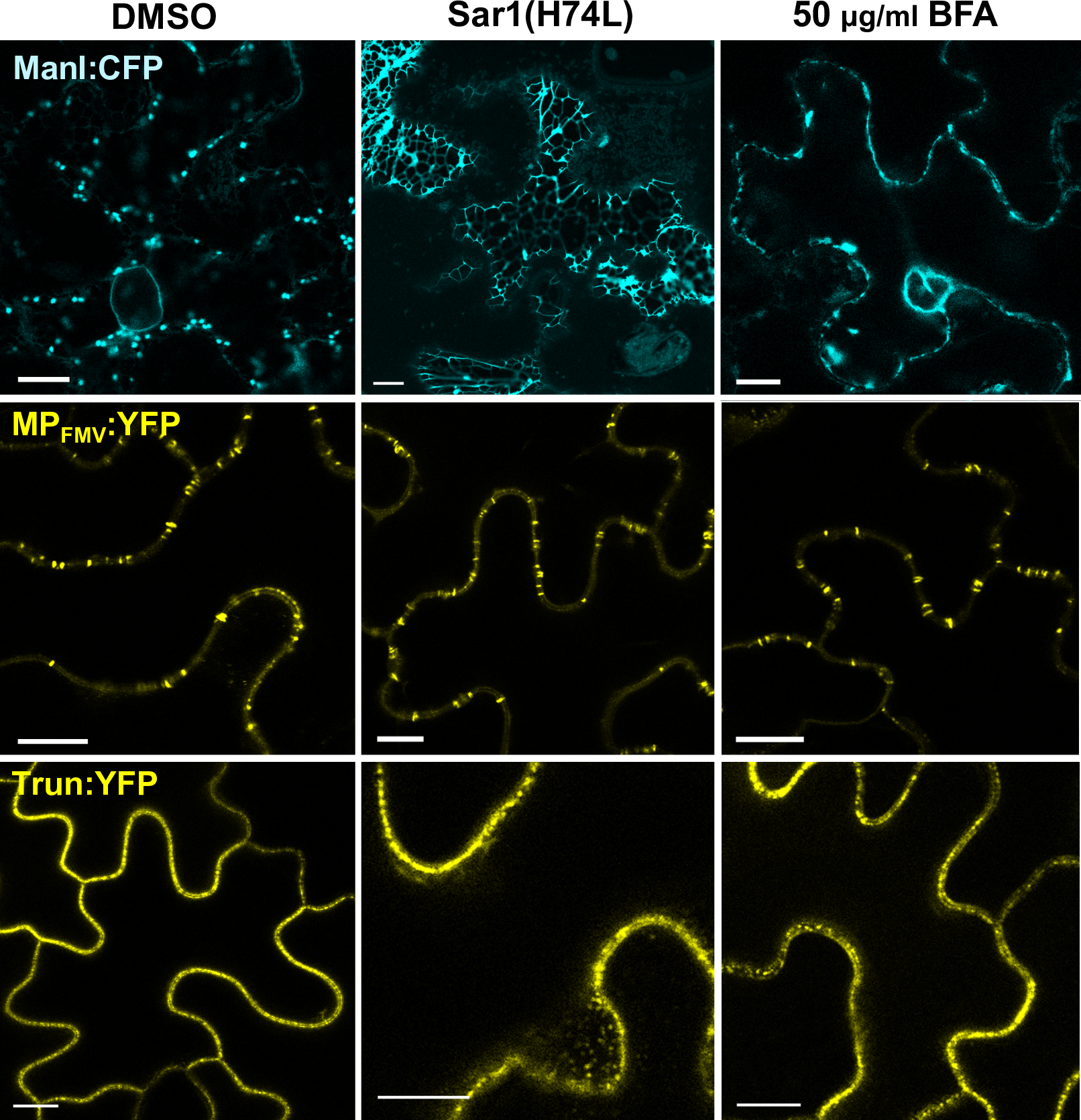

Supplement: S8 Fig — Whether COPII transport is involved in the localization of MPFMV:YFP and Trun:YFP was tested by treatments with 0.5%(v/v) dimethyl sulfoxide (DMSO), 50 μg/ml brefeldin A (BFA) or expression of Sar1(H74L). A Golgi marker, ManI:CFP was used as a control. Cells were observed at 24 hpi. Bars = 10 μm. (TIF) [file ppat.1006463.s008.tif]
